# Innovation of eco-friendly TiO2 nano catalyst for new pyrimidine carbonitiriles candidates, assessed for significant antioxidant activity, anti-inflammatory effects, and by insilico studies

**DOI:** 10.1371/journal.pone.0313959

**Published:** 2025-05-29

**Authors:** Wesam S. Shehab, Ahmed F. EL-Farargy, Abdel Rahman B. A. El-Gazzar, Hend A. Haikal, Samar M. Mouneir, Abdul-Hamid M. Emwas, Mariusz Jaremko, Magda H. Abdellattif

**Affiliations:** 1 Department of Chemistry, Faculty of Science, Zagazig University, Zagazig, Egypt; 2 Department of Organic Chemistry, National Research Centre, Dokki, Cairo, Egypt; 3 Department of Pharmacology, Faculty of Veterinary Medicine, Cairo University, Cairo, Egypt; 4 Core Labs, King Abdullah University of Science and Technology (KAUST), Thuwal, Saudi Arabia; 5 Biological and Environmental Science and Engineering (BESE), King Abdullah University of Science and Technology (KAUST), Thuwal, Saudi Arabia; 6 Department of Chemistry, Sciences College, University College of Taraba, Taif University, Taif, Saudi Arabia; University of Mashreq, IRAQ

## Abstract

Production of titanium dioxide nanoparticles (TiO_2_- NPs) is carried out in high quantities for an extensive range of applications. These nanoparticles have various physicochemical properties, which can affect their bioactivity. The Biginelli synthesis of 4-cyanobenzaldehyde, ethyl cyanoacetate, and thiourea in the presence of TiO_2_ nanoparticle yields 4-(4-cyanophenyl)-6-oxo-2-thioxohexahydropyrimidine-5-carbonitrile **(1)**. This multi-component system is safe, eco-friendly, and non-toxic. The introduction of the new system saves time and reduces chemical usage. Compound **(1)** underwent reactions with various additional compounds such as (methyl iodide, chloroacetonitrile, chloroacetone, acrylonitrile, ethyl chloroacetate, and chloroacetic acid/benzaldehyde). The newly synthesized compounds possess remarkable anti-inflammatory and antioxidant activities, making them an excellent choice for therapeutic use.. The in-silico studies include molecular docking using MOE, Pharmacokinetics using Toxoradar, SAR, and DFT studies.

## Introduction

The rise of nanotechnology and eco-friendly chemical synthesis has revolutionized the use of nanoparticles in a multitude of industries. From producing antibacterial materials to enhancing drug delivery systems and creating sunblock to perfecting makeup and microchip technology, nanoparticles have become vital in achieving optimal results. As pyrimidine is a ring involved in DNA and RNA, this indicates higher pharmacological activities; some of the most significant are anticancer, antiviral, particularly anti-HIV, antimicrobial, anti-inflammatory, and antioxidant activities [1–11]. Creating new candidates of pyrimidine thione derivatives with potential pharmacological effects is one of our aims. Regarding heterogeneous catalysis, the catalyst recovery and separation from the reaction matrix were considered by utilizing various supporting catalysts to restrain the particle and provide a suitable surface area. [12]. The Biginelli synthesis, which produces pyrimidine compounds, has been extensively studied in recent decades, particularly because of the therapeutic properties of the resulting compounds, which include calcium channel blockers, anticancer, antiviral, antimicrobial, anti-inflammatory, and antioxidant compounds [13–14]**. This approach creates a stable and solid framework for the heterogeneous catalyst, which is not soluble in the solution matrix.** [15].

Due to cost-effective synthesis modes, various heterogeneous catalysts such as TiO_2_, Al_2_O_3_, ZrO_2_, and ZnO are being manufactured and have become widely available. For example, TiO_2_ is a heterogeneous catalyst used in fuel processing due to its tunable porous surface, high thermal stability, and mechanical strength. [16]. On the other hand, the pharmaceutical industry has resorted to several strategies to improve these compounds for use as drugs. DPPH is a free radical that may be used to determine radical scavenging and related antioxidant activities [17]. The antioxidants help prevent and reduce damage from free-radical reactions because of their ability to donate electrons that can neutralize the formation of free radicals [4,18,19]. Inflammation is a reaction to injury of living tissues, comprising systemic and local responses [20]. Inflammation is also a cellular reaction to harmful stimuli and infections. This physiological response includes delivering blood components to the local site of infection or injury, triggering vasodilation and increased vascular permeability. While inflammation is a healing response of the living body, the body cannot continuously regulate this response, and inflammatory excess can threaten the body. Thus, by understanding how inflammation functions, we may better understand its significance in maintaining our health while helping us discover new anti-inflammatory therapeutics. The hemolytic method is well-known in the biological assessment of anti-inflammatory compounds [21,22], and using the hemolytic and other techniques, the present paper reports the synthetization and characterization of pyrimidine carbonitrile derivatives, which are known as good, dual nature antioxidant and anti-inflammatory drugs. Molecular docking within a molecular operating environment MOE (2019), DFT with Spartan 20, and SAR (structure-activity relationship and pharmacokinetics) are also applied using Molinspiration and Toxiradar [23–25].

## 2. Results and discussion

### 2.1. Nanoparticles titanium dioxide

**Fig 1:**
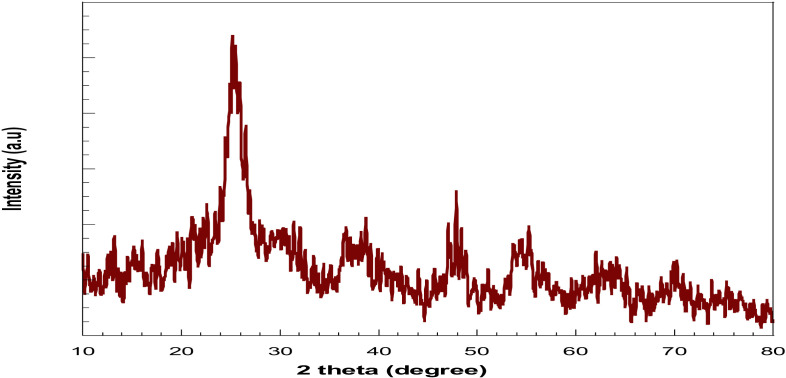
XRD pattern of TiO_2._

Fig 1 represents the XRD pattern of the produced nano-TiO_2_, revealing diffraction peaks at 2θ =  25 and 48°; TiO2 was recorded in the anatase phase. [26]. The peaks are in an upright position in agreement with the standard spectrum (JCPDS № 01-089-4921); one can notice that the intensity of the diffraction pattern of the TiO2 is directly proportional to the reduction of the particle size. This result indicates the formation of the nano-TiO_2_ [27]. At the same time, the expected diffraction signal of the rutile phase at 27.5 ^o^ was not detected, which indicated that the formed TiO_2_ was only of a particular anatase phase. [28].

**Fig 2:**
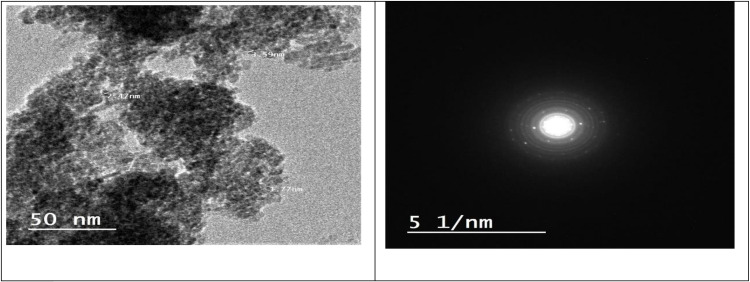
TEM micrograph and SAED of TiO_2._

Ultrastructural imaging via transmission electron microscopy (TEM) was used to examine the nanocrystals’ crystalline phases, size, and aggregation. The TEM images of the catalysts confirmed that the TiO_2_ comprises combinations of mostly spherical nanoparticles with an average diameter of ∼5 nm. Therefore, TEM was used to supplement the examination of the particle size, crystallinity, and morphology of the TiO_2._ [29]; Fig 2 shows TEM images of TiO2 in anatase phases. The area electron diffraction (SAED) patterns of the nano TiO2 powder display typical rings that match the anatase phase. The brightness and intensity of the call signals are weak, meaning that TiO_2_ was poorly crystallized and partially amorphous.

### 2.2 Chemistry

**Scheme 1:**
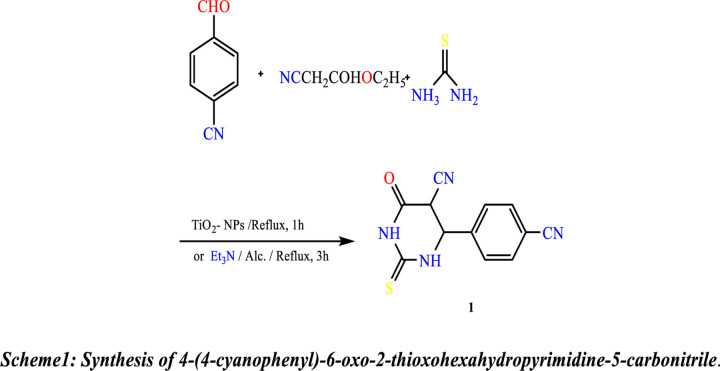
Synthesis of 4-(4-cyanophenyl)-6-oxo-2-thioxohexahydropyrimidine-5-carbonitrile.

**Scheme 2:**
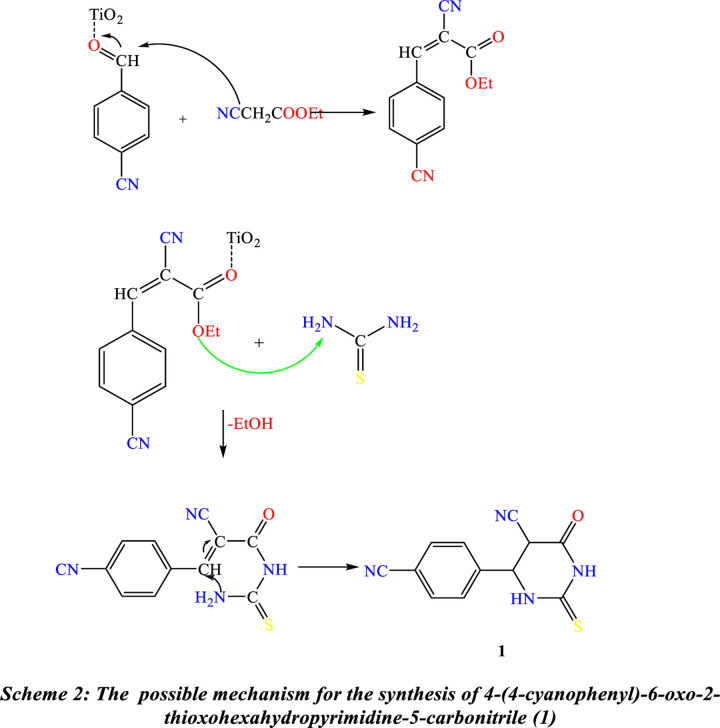
The possible mechanism of the synthesis of 4-(4-cyanophenyl)-6-oxo-2-thioxohexahydropyrimidine-5-carbonitrile (1).

Synthesis of new heterocyclic molecules was carried out depending on the pyrimidine moiety. The starting material 4-(4-cyanophenyl)-6-oxo-2-thioxohexahydropyrimidine-5-carbonitrile **(1)** was produced using two methods. The optimized reaction conditions are represented in Table 1. The traditional method involves the reaction of thiourea and ethyl cyanoacetate with 4-cyanobenzaldehyde in the presence of a few drops of triethylamine in ethanol under reflux conditions. The reaction of the three components of the TiO_2_ nanoparticles (TiO_2_-NPs) as a catalytic amount in ethanol under reflux was archived to obtain Compound **(1)** with optimal reaction conditions (Scheme 1). Infrared (IR), proton, and carbon-13 nuclear magnetic resonance (^1^H-NMR and ^13^C-NMR) were used to elucidate Structure **(1)**. The IR spectra showed the presence of a C = S band at 1270 cm^-1^, an absorption band at 1721 cm^-1,^ which is due to the C = O group, an absorption band at 2224 cm^-1^ due to the CN group, and an absorption band over the range cm^-1^ attributed to the 2NH groups. Due to the CH aromatic, the ^1^H NMR spectra show two doublet signals at 8.05 and 8.15. The spectra also show two singlet signals at 8.17 and 8.49 due to NH groups, whereas the ^13^C NMR spectrum shows two signals at (^δ^ᵹ in ppm) 115.06, 118.14 due to 2 CN groups, a signal at 161.22 due to C = O group and a signal due to C = S group at 183.81 (Scheme 2).

**Table 1:** Optimized reaction conditions.

Compound	Solvent	Catalyst (base)	Temperature (°C)	Time (h)	Yield (%)
1	Ethanol	TEA	Reflux	3	35
1	Ethanol	TiO_2_	Reflux	1	40
2	Ethanol	NaOEt	Reflux	3	60
2	Ethanol	TiO_2_	Reflux	2	72
5	Ethanol	Pyridine	Reflux	5	45
5	Ethanol	TiO_2_	Reflux	3	57

**Scheme 3:**
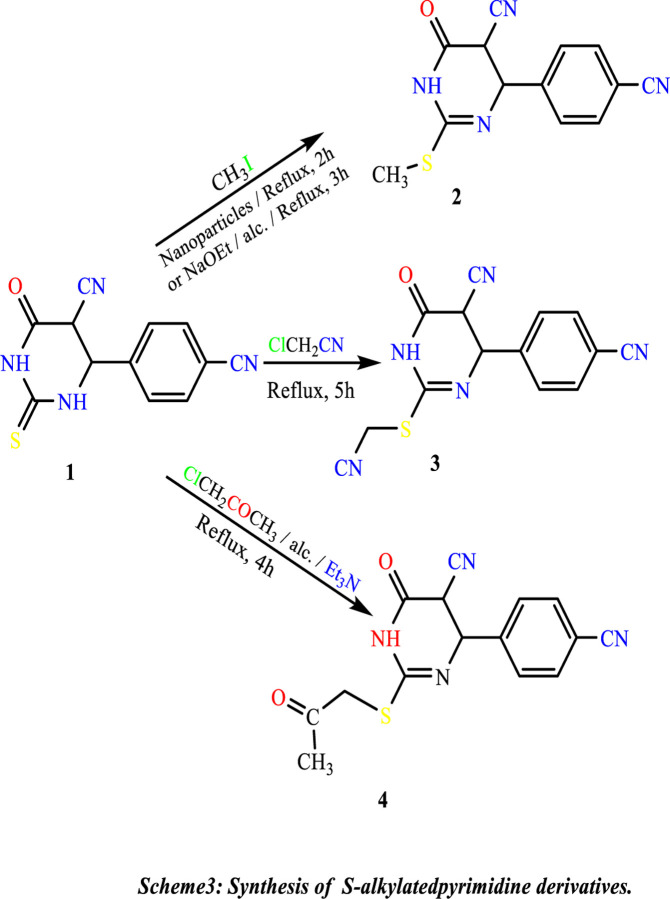
Synthesis of S-alkylatedpyrimidine derivates.

The introduction of a good leaving group was performed by treatment of **(1)** with methyl iodide in ethanolic sodium ethoxide, leading to the formation of the methyl-thio derivative **(2)**. Also, the alkylation reaction is carried out using TiO_2_-NPs as an essential environment, while alkylation (cyanomethylation) with chloro-acetonitrile in ethanolic sodium ethoxide gave 2-((cyanomethyl)thio)-4-(4-cyanophenyl)-6-oxo-1,4,5,6-tetrahydropyrimidine-5-carbonitrile **(3)**. Alkylation of Compound **(1)** with chloroacetone under primary conditions gave 4-(4-cyanophenyl)-6-oxo-2-((2-isopropyl)thio)-1,4,5,6-tetrahydropyrimidine-5-carbonitrile **(4) (**Scheme 3).

**Scheme 4:**
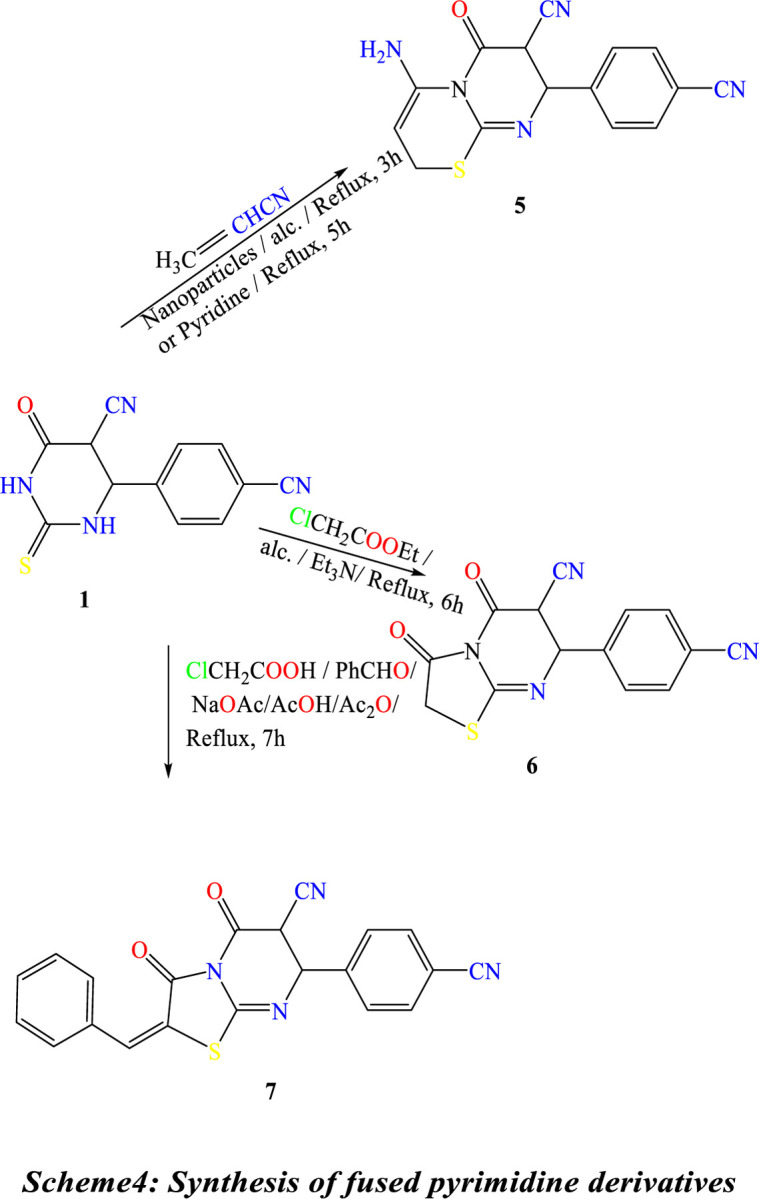
Synthesis of fused pyrimidine derivatives.

**Scheme 5:**
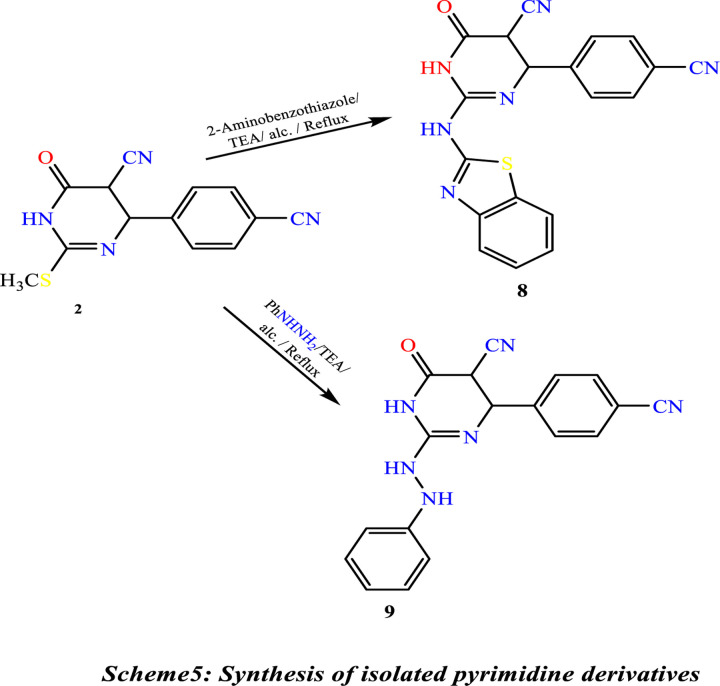
Synthesis of isolated pyrimidine derivatives.

Treatment of **(1)** with acrylonitrile in pyridine gave 4-amino-8-(4-cyanophenyl)-6-oxo-7,8-dihydro-2H,6H-pyrimido[2,1-b][1,3]thiazine-7-carbonitrile **(5)**. Compound **(5)** is also produced through the reaction of thiol Compound **(1)** by Cyanomethylation with acrylonitrile in the presence of TiO_2_-NPs as a catalytic amount in ethanol. The reaction of Compound **(1)** with ethyl chloroacetate under primary conditions gave the thiazolo[3,2-a]pyrimidine-6-carbonitrile derivative **(6)**. In contrast, the reaction of Compound **(1)** with chloroacetic acid and benzaldehyde in the presence of sodium acetate under reflux in a mixture of glacial acetic acid and acetic anhydride gave the benzylidene derivative ofthiazolo[3,2-a]pyrimidine-6-carbonitrile **(7)**, (Scheme 4). The structures of Compounds **(5)**, **(6)**, and **(7)** were confirmed based on their spectral data (experimental part). A shred of evidence in confirmation of the structure of **(2)** is its conversion into 2-(benzo[d]thiazol-2-ylamino)-4-(4-cyanophenyl)-6-oxo-1,4,5,6-tetrahydropyrimidine-5-carbonitrile **(8)** by reaction with 2-amino benzothiazole in the presence of few drops of triethylamine and also its conversion into 4-(4-cyanophenyl)-6-oxo-2-(2-phenylhydrazine)-1,4,5,6-tetrahydropyrimidine-5-carbonitrile **(9)** by reaction with phenyl-hydrazine under similar condition, (Scheme 5). ^1^H NMR spectra indicate the disappearance of the signal of the S-CH_3_ methyl group of Compound 2 at ᵹ 2.53 ppm. Compound **2** was obtained by reaction of 2-amino benzothiazole with a few drops of triethylamineafforded2-(benzo[d]thiazol-2-ylamino)-4-(4-cyanophenyl)-6-oxo-1,4,5,6-tetrahydropyrimidine-5-carbonitrile **(8).** Under similar experimental conditions, Compound **(2)** was reacted with phenyl-hydrazine to yield 4-(4-cyanophenyl)-6-oxo-2-(2-phenylhydrazine)-1,4,5,6-tetrahydropyrimidine-5-carbonitrile **(9) (**Scheme 5**).**

### 2.3 Biology

#### 2.3.a Antioxidant activity.

**Fig 3:**
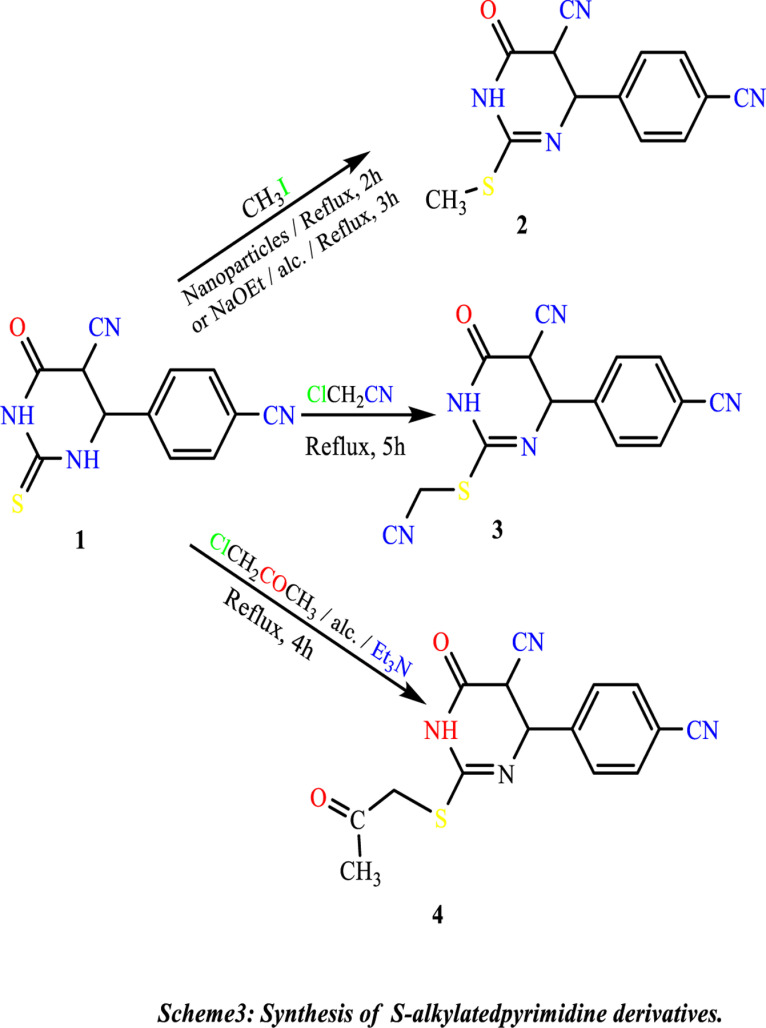
Antioxidant activity by DPPH radical scavenging method.

The antioxidant activity of the newly synthesized compounds was evaluated using DPPH (2, 2-diphenyl-1-picrylhydrazyl (DPPH) assay). This assay measures the ability of a compound to neutralize the stable free radical DPPH by donating an electron or hydrogen atom. The results are illustrated in Table 2 and Fig 3. All the tests and analyses were undertaken in three replicates, and the results were averaged. All the tested compounds exhibited antioxidant activity. Compounds **(5)**, **(8)**, and **(2)** showed high activity with IC_50_ 5.62, 6.21, and 10.09 μg/ml compared to 4.63 of standard ascorbic acid. Compounds **(1**), **(4**), and **(3)** showed moderate activity with IC_50_ 25.13, 35.31, and 35.9, respectively.

**Table 2:** Antioxidant activity of the synthesized compounds by using DPPH scavenging%.

Compound	IC_50_ug/ml Molecular wt(g/mol) IC_50_ μM
Ascorbic acid	**4.63 176.12 26.289**
1	25.13 **256.28 98.06**
2	10.09 **270.06 37.03**
3	35.9 **295.32 121.56**
4	35.31 **312.35 113.05**
5	5.62 **309.35 18.17**
6	46.69 296.30 173.37
8	6.21 372.08 16.69
9	212.28 330.35 642.59

The mechanism of antioxidant activity may be explained by inhibiting lipid peroxidation in addition to cyclooxygenase and lipo-oxygenase catalysis [29]. Also, previous studies [30,31] report that pyrimidine derivatives show antioxidant activity by DPPH scavenging ability. In addition, the incorporation of titanium dioxide nano-catalysts in synthesizing pyrimidine carbonitrile derivatives plays a crucial role in enhancing the antioxidant activity of the newly synthesized compounds. Titanium dioxide nano-catalysts can act as free radical scavengers and facilitate the regeneration of antioxidant compounds. It also modulates the cellular signaling pathway involved in oxidative stress and inflammation by enhancing superoxide dismutase, catalase, and glutathione peroxidase. Adding titanium dioxide nano-catalyst may enhance *in vivo* antioxidant activity through suppression of genes involved in apoptosis and immune response as NF-Kβ, FGFR2, and Casp8 and boosting pentose-phosphate, cysteine methionine, glycine-serine metabolism pathway [32].

#### 2.3.b Anti-inflammatory activity.

**Fig 4:**
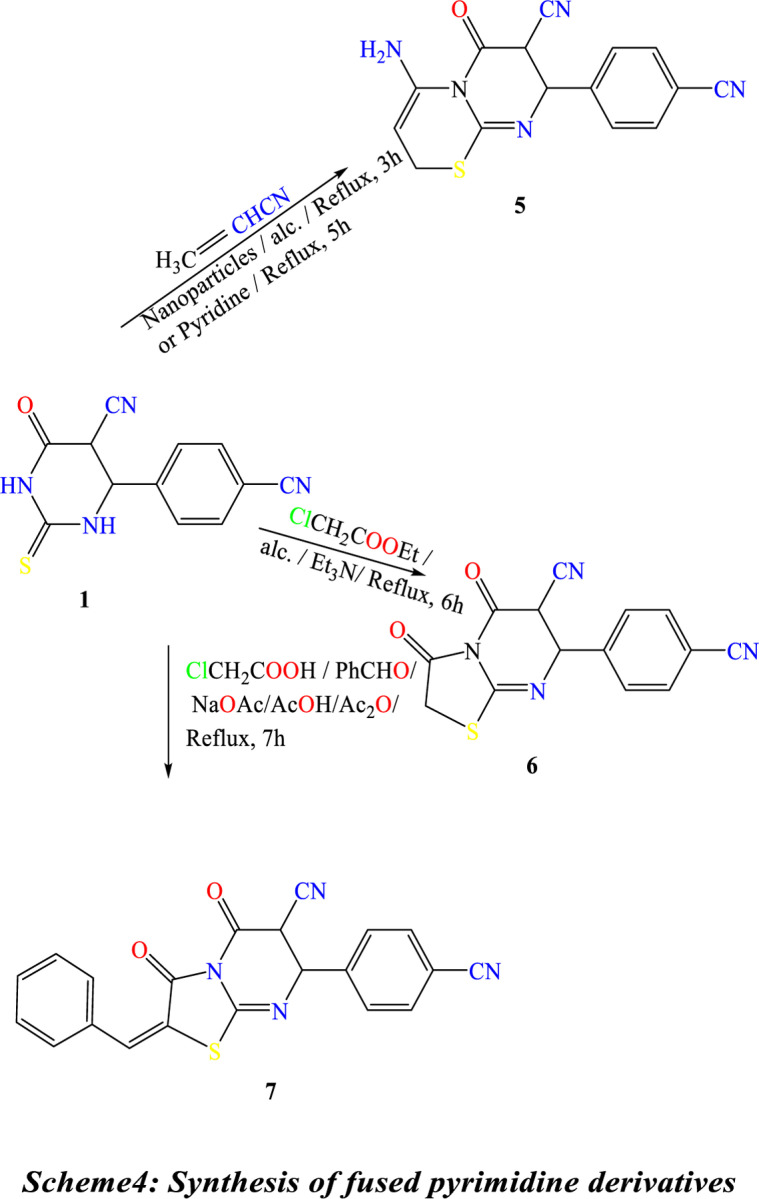
Anti-inflammatory activity of the tested compounds.

The hemolysis essay of the synthesized compounds is summarized in Table 3 and Fig 4; the mean absorption rate of the samples, with isotonic solution Ab, and hemolysis % were calculated with a standard deviation. HRBC and membrane stabilization assay results provide valuable information about the potential anti-inflammatory effects of the newly synthesized pyrimidine carbonitrile derivatives. All the tested compounds showed potent anti-inflammatory activity. The most powerful compounds were **(2)** and **(8)**, as there was no hemolysis when rheu was used at 100 μg/ml, followed by Compound **(5)**, which exhibited 0.2% hemolysis when used at the same concentration. The results for the anti-inflammatory activity are in agreement and homogenous with those obtained from the antioxidant activity of the same compounds, as shown in Table 3. The interconnection between inflammation and oxidative stress plays a vital role in the induction of several chronic diseases, such as diabetes, rheumatoid arthritis, and inflammatory bowel disease—inflammation results in the release of inflammatory mediators and, subsequently, tissue damage. Oxidative stress occurs due to an imbalance between the production of the reactive oxygen species and the ability of the body to neutralize them. Several cellular mechanisms are involved in the anti-inflammatory activity of the tested compounds. A previous study [33] reports that pyrimidine, as a heterocyclic compound, shows potent anti-inflammatory activity in carrageenan-induced rat paw edema by inhibiting the inflammatory mediator PGE 2 via the inhibitory activity of cyclooxygenase enzymes. The cellular mechanism of action was detected by suppression of myeloperoxidase and granulocyte superoxide formation. These compounds also inhibit the expression of TNF, nuclear factor kβ, interleukins, and leukotrienes. Another *in vivo* study [34] explains that some pyrimidine derivatives possess anti-inflammatory activity greater than both standard celecoxib and diclofenac sodium in both formalin-induced paw edema and cotton pellet granuloma in rats in acute and chronic inflammation models. In addition, other mechanisms may explain the anti-inflammatory activity through the inhibitory effect on nitric acid synthase and lipo-oxygenase enzymes [35,36].

**Table 3:** Anti-inflammatory activity by hemolysis.

Conc. ug/ml	Ab. Mean of Sample with RBCs	Sample with Isotonic solution Ab.	Hemolysis %	SD.
**Control Complete Hemolysis**	**1.693**	**0.002**	**100**	**0.002**
**Compound 1**				
1000	0.417	0.181	13.9	0.004
800	0.358	0.134	13.2	0.003
600	0.302	0.092	12.4	0.003
400	0.269	0.065	12.0	0.006
200	0.195	0.035	9.5	0.003
100	0.144	0.018	7.5	0.005
0	0.000	0.001	0.0	0.001
**Compound 2**				
1000	0.657	0.62	2.2	0.008
800	0.579	0.574	0.3	0.010
600	0.421	0.416	0.3	0.001
400	0.343	0.341	0.1	0.001
200	0.280	0.278	0.1	0.002
100	0.147	0.147	0.0	0.003
0	0.000	0.001	0.0	0.001
**Compound 3**				
1000	1.693	0.002	100	0.002
800	0.348	0.309	2.3	0.004
600	0.239	0.218	1.2	0.003
400	0.173	0.161	0.7	0.002
200	0.091	0.085	0.3	0.002
100	0.044	0.043	0.1	0.002
0	0.029	0.027	0.1	0.001
**Compound 4**				
1000	0.281	0.123	9.3	0.002
800	0.218	0.087	7.8	0.002
600	0.161	0.062	5.9	0.006
400	0.102	0.041	3.6	0.003
200	0.055	0.024	1.8	0.002
100	0.034	0.011	1.3	0.002
0	0.000	0.001	0.0	0.001
**Compound 5**				
1000	0.462	0.344	7.0	0.009
800	0.378	0.278	5.9	0.003
600	0.328	0.235	5.5	0.005
400	0.265	0.241	1.4	0.002
200	0.156	0.15	0.4	0.002
100	0.101	0.098	0.2	0.005
0	0.000	0.001	0.0	0.001
**Compound 6**				
1000	0.367	0.125	14.3	0.002
800	0.312	0.095	12.8	0.003
600	0.148	0.071	4.6	0.111
400	0.109	0.041	4.0	0.002
200	0.084	0.032	3.1	0.002
100	0.060	0.025	2.1	0.005
0	0.000	0.001	0.0	0.001
**Compound 8**				
1000	0.638	0.575	3.7	0.020
800	0.444	0.394	3.0	0.003
600	0.300	0.278	1.3	0.002
400	0.185	0.169	0.9	0.004
200	0.117	0.115	0.1	0.002
100	0.093	0.093	0.0	0.002
0	0.000	0.001	0.0	0.001
**Compound 9**				
1000	0.485	0.241	14.4	0.007
800	0.302	0.133	10.0	0.004
600	0.244	0.092	9.0	0.001
400	0.149	0.064	5.0	0.003
200	0.117	0.045	4.3	0.002
100	0.070	0.022	2.9	0.002
0	0.000	0.001	0.0	0.001

### 2.4 In-silico-studies

#### 2.4.a Molecular docking.

##### 2.4.a.1 Anti-inflammatory activity

**Fig 5:**
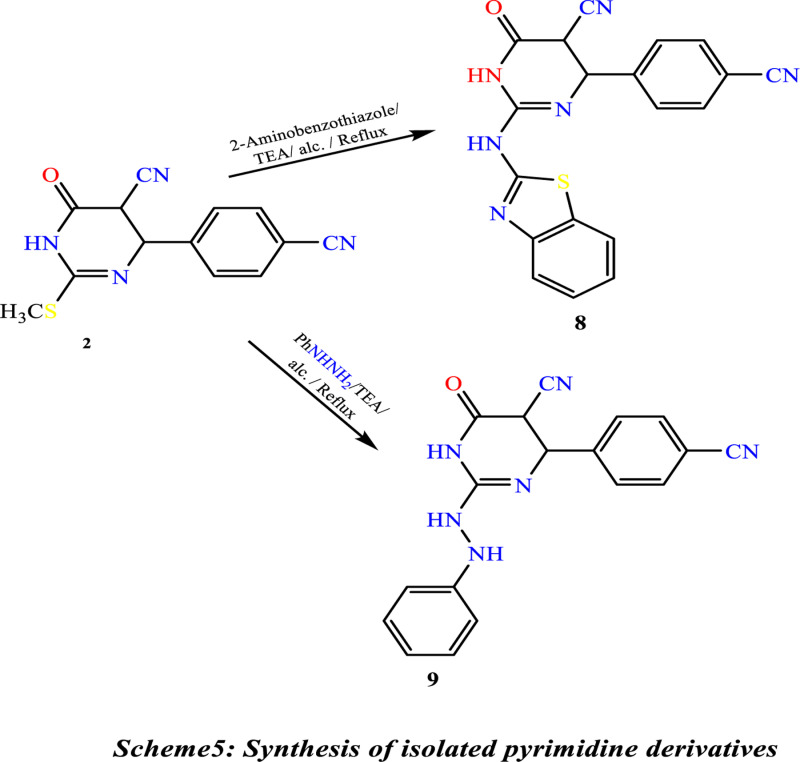
shows the selective COX-2 inhibitor PDB: 1CX2,.

To acquire insights into the underlying mechanisms of action of the newly synthesized pyrimidine carbonitrile derivatives, we carried out virtual docking of the highly selective COX-2 inhibitors with Compounds **5** and **8** within the active binding site of the COX-2 enzyme. The co-crystal structure of the COX-2 complex with SC-558, a selective COX-2 blocker, was acquired from the PDB (protein data bank) **PDB: 1CX2.** Fig 5 indicates the 2D and 3D snap of the interacting compounds with receptors. The docked co-crystal is used for validation and shows docking score energy with ‐7.23 kcal/mole with rmsd of 1.556, the docked co-crystal and the PDB **1CX2** showed interaction at ARG 469 (A) H-donor, SER 471 (A) H-acceptor, and GLN 543 (B) pi-H. The docked complexes **5** and **8** showed docking score energy expressed in kcal/mol with -6.45, and ‐7.4099, with rmsd values 0.8358 and 1.0393 respectively. Docked complex **5** showed interactions with TYR 122 (A) H-donor and ILE 124 (A) H-donor. Docked complex **8** showed interactions at LYS 468 (A) H-donor, THR 62 (A) H-acceptor, GLY 63 (A) H-acceptor, and LYS 83 (A) pi-cation.

##### 2.4.a.2. Antioxidant activity

**Fig 6:**
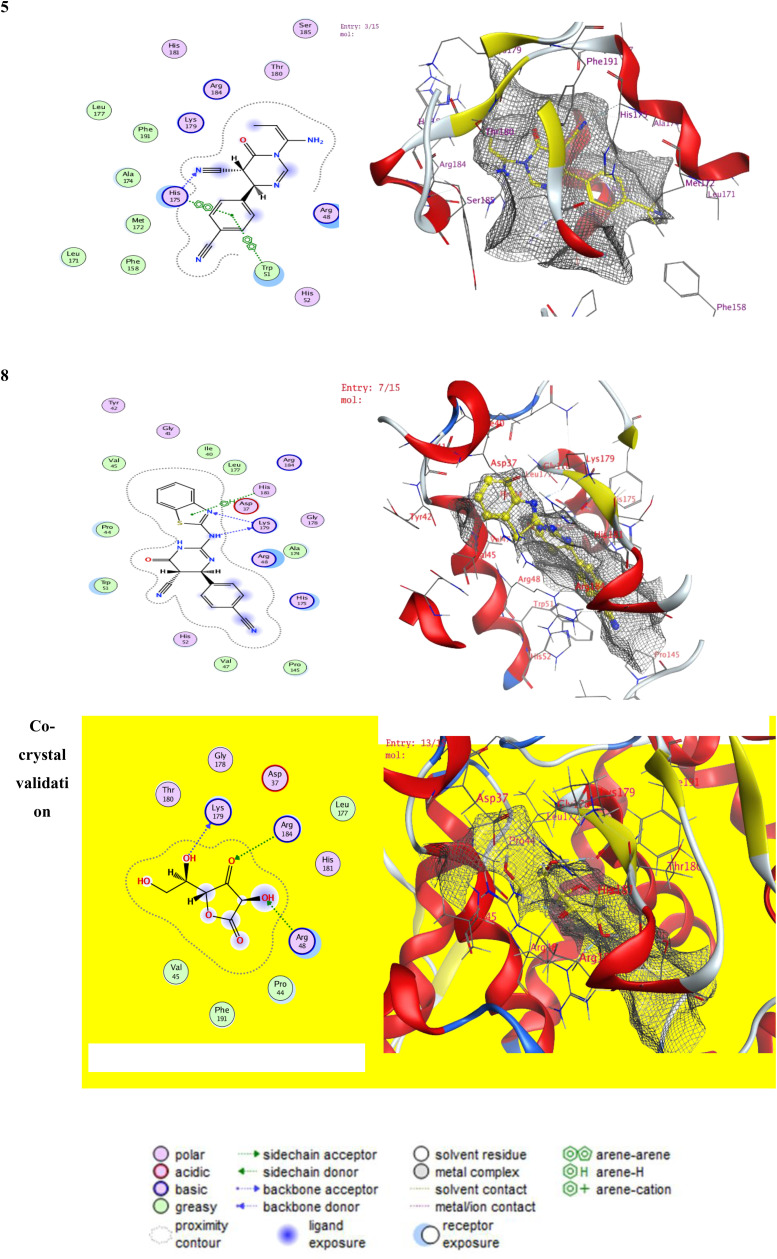
The interaction of *c* peroxidase enzyme PDB: 2X08.

A molecular docking study of the newly synthesized pyrimidine carbonitrile derivatives was carried out on Compounds **(5)** and **(8)** using co-crystallized ascorbic acid as a reference ligand, isolated from cytochrome *c* peroxidase enzyme **PDB: 2X08**. The docked co-crystal is used for validation and shows docking score energy with -4.93 kcal/mole with rmsd of 0.564, the docked co-crystal and the PDB **2X08** showed interaction at LYS 179 (A), with H- donner, ARG 48 (A) H-acceptor, and ARG 184 (A) H-acceptor. The docked complexes 5 and 8 showed docking score energy with -7.005, and -7.89, with rmsd values 1.21 and 1.29 respectively. The results are represented in Fig 6, indicating the 2D and 3D snap of the interactive compounds with receptors.. Docked complex **5** showed interactions with HIS 175 (A) H-acceptor, HIS 175 (A) pi-pi, TRP 51 (A) pi-pi, and Arg84 by ligand exposure with binding to the γ-heme edge of cytochrome c peroxidase. Docked complex **8** showed interactions at LYS 179 (A) H-donor, LYS 179 (A) H-acceptor, and HIS 181 (A) pi-H. at the same time, the docked co-crystal showed interactions at LYS 179 (A) H-donor, ARG 48 (A) H-acceptor, and ARG 184 (A) H-acceptor.

#### 2.4.b Pharmacokinetics.

##### ProTox-II

ProtoxII virtual lab is used to predict the toxicities of small molecules.

**Fig 7:**
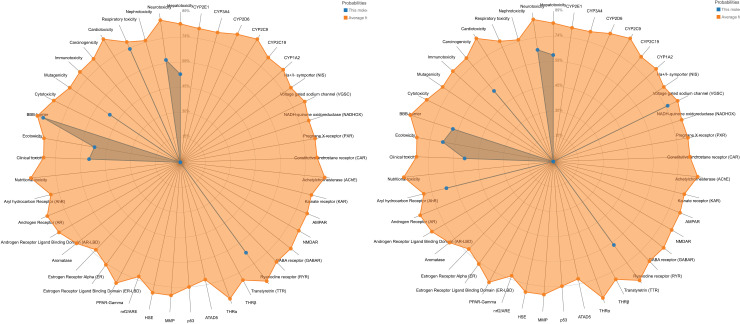
The toxicity radar chart is intended to quickly illustrate the confidence of positive toxicity results compared to the average of its class for Compounds (5) and (8).

Predicting compound toxicities is an integral part of the drug design development process. The ProTox-II shows that the four compounds are expected to have oral LD50 values ranging from 159 to 2480 mg/kg in a rat model with (1 s,4 s)-eucalyptol having the highest values and quercetin the lowest. The toxicity radar Fig 7 is intended to quickly illustrate the confidence of positive toxicity results compared to the average of its class for Compounds **(5)** and **(8**) (Table 4).

**Table 4:** The predicted toxicity for (5) and (8) using (a) the ProTox-II and (b) the Pred-hERG software.

	5	8
a-**Pro-ToxII**		
**Predicted LD50 (mg/kg)**	700 mg/kg	1000 mg/kg
**Predicted toxicity class**	4	
**Average similarity (%)**	54.72%	54.01%
**Prediction accuracy (%)**	67.38%	67.38%
b-**Pred-hERG**		
**Prediction/Potency**	Weak or Moderate	Weak or Moderate
**Confidence (%)**	60	60
**Applicability domain (AD.)**	No (Value = 0.25 and limit = 0.26)	No (Value = 0.28 and limit = 0.26)

##### Pred-hERG

**Fig 8:**
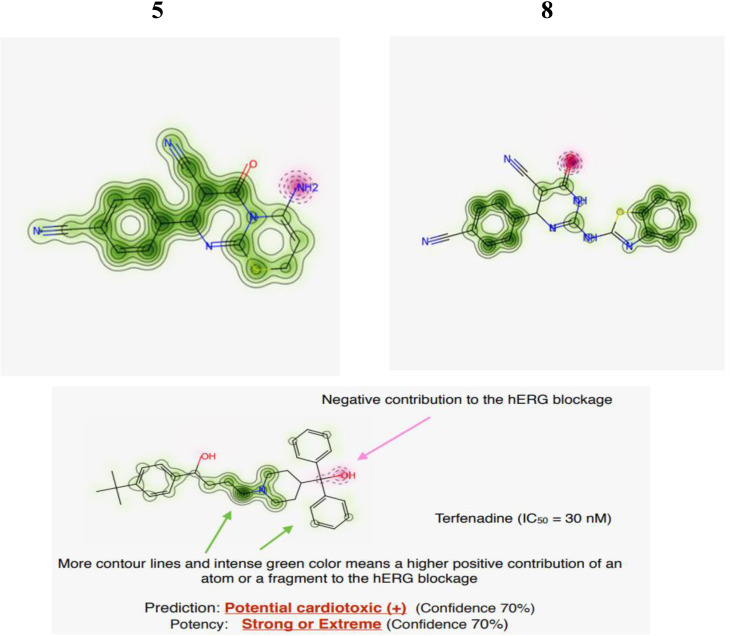
Pred-hERG results. Probability map of Compounds (5) and (8).

**Fig 9:**
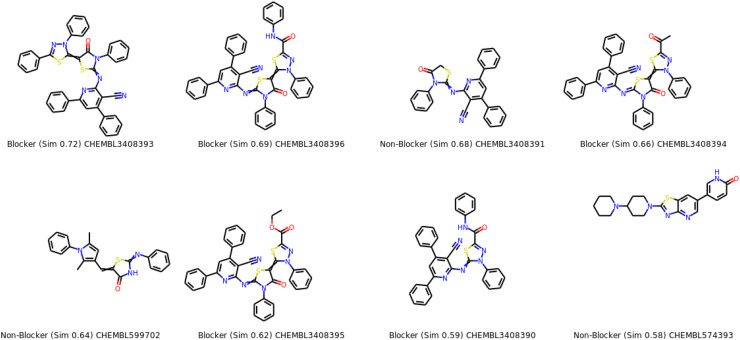
compound 5.

**Fig 10:**
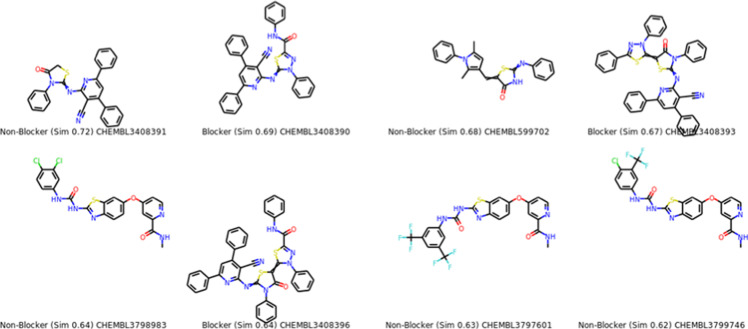
compound 8.

Chemically similar compounds can have different effects on different protein targets, and proteins may not always be able to distinguish between similar ligands. To improve machine learning predictions, knowledge of pharmacological and off-target relationships between proteins and the similarity of ligand sets can be used to interpolate outputs and ensure that predictions are consistent with compound similarity criteria. This pipeline helps to improve the forecasts of off-target drug effects and reduce the false-negative error. Chemical similarity is one of the essential concepts in cheminformatics. One commonly used to calculate these similarity algorithm measures is the 2D Tanimoto algorithm employed here. The resulting Tanimoto coefficient is fingerprint-based, encoding each molecule to a fingerprint “bit” position (MACCS), with each bit recording the presence (“1”) or absence (“0”) of a fragment of the molecule. The potency results are represented in Table 5b, while Fig 8 shows the probability mp of Compounds **(5)** and **(8)**. The similar off-compounds of Compounds **(5)** and **(8)** are represented in Fig 9 **and** Fig 10.

**Table 5:** Physicochemical properties of the synthesized compounds.

Compound	miLogP		TPSA		n-atoms	MW.		nON	nOHNH		n-violations		n-rotb		Volume
**5**	0.31		106.28		22	309.35		6	2		0		1		258.40
**8**	2.72		113.97		27	372.41		7	2		0		0		309.58
**b-Physicochemical Molinspiration bioactivity score**															
**Compound**		**GPCR ligand**		**Ion channel modulator**			**Kinase inhibitor**			**Nuclear receptor ligand**		**Protease inhibitor**		**Enzyme inhibitor**	
**5**		-0.51		-0.72			-0.81			-0.99		-0.35		-0.44	
**8**		-0.29		-0.44			-0.42			-0.81		-0.26		-0.29	

##### Similar off-compounds induced by labMol for Molinsipration

The predicted pharmacokinetic/Molinspiration properties of the pyrimidine carbonitrile derivatives **(5)** and **(8)** are given in Table 5a. With the help of Molinspiration virtual screening, most synthesized compounds showed promising bioactivity as implied by docking parameters in Table 5b, which indicates the drug-likeness properties against kinase inhibitors, protease, and enzyme inhibitors. The calculated distribution of activity scores (version 2011.06), GPCR ligands, kinase inhibitors, ion channel modulators, nuclear receptor ligands, protease inhibitors, and other enzyme targets are compared with scores for about 100,000 average drug-like molecules. The score allows efficient separation of active and inactive molecules.

#### 2.4.c. DFT.

**Fig 11:**
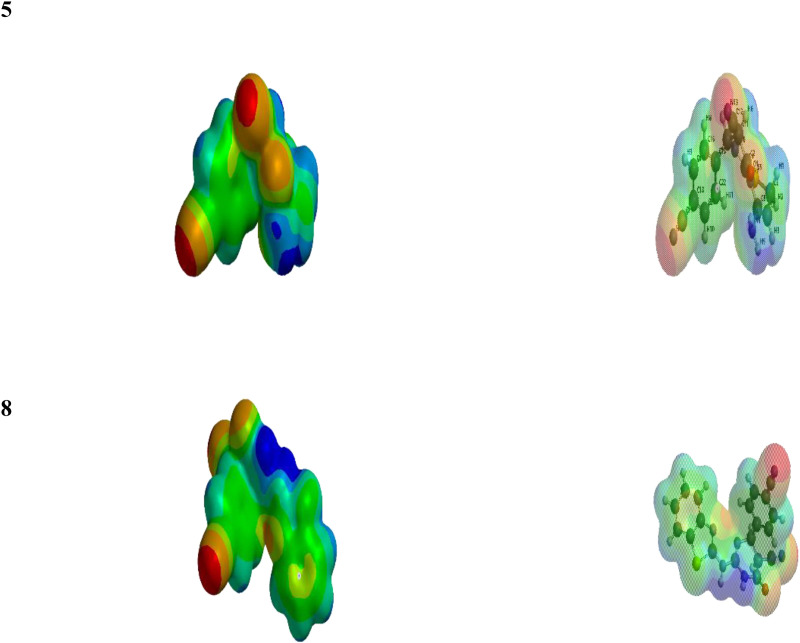
Electrostatic potential map of Compounds (5) and (8).

**Fig 12:**
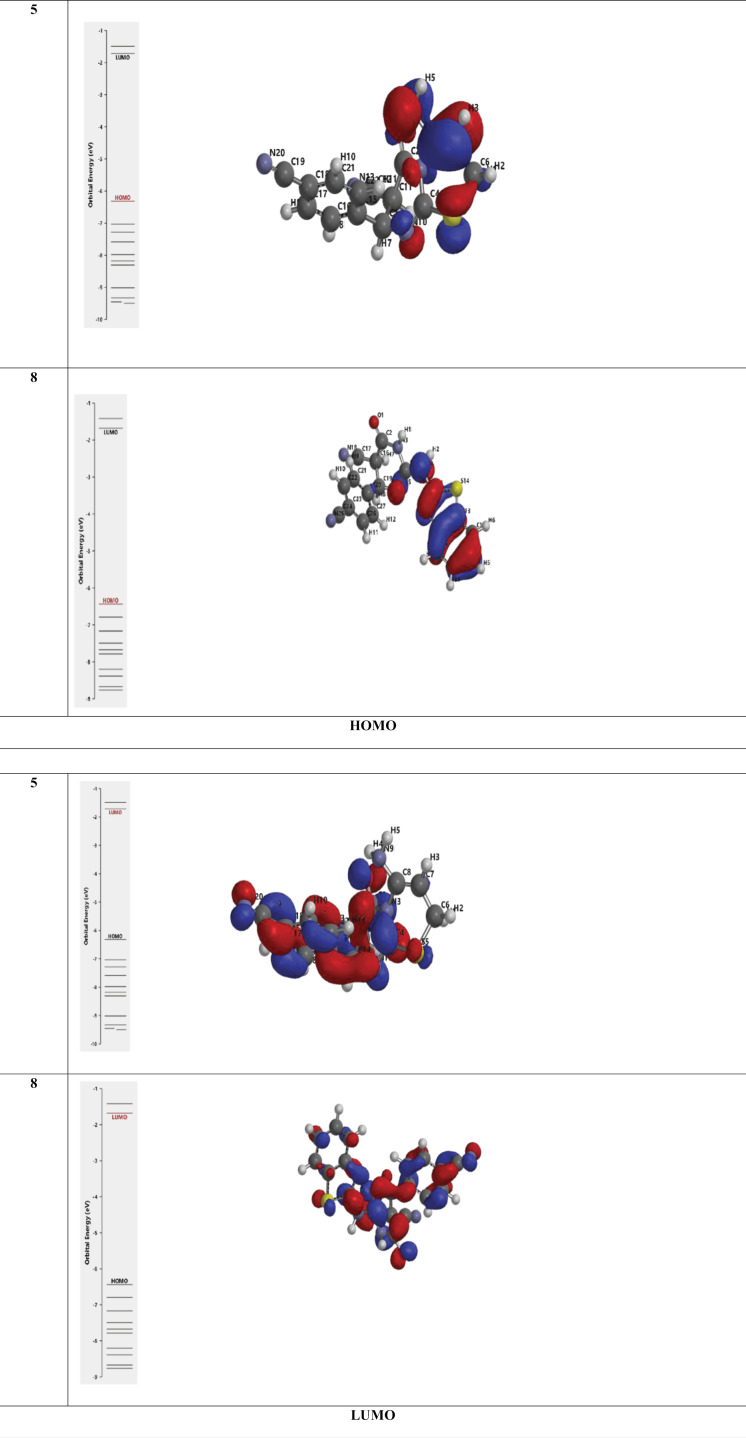
The optimized structure and its HOMO and LUMO of Compounds (5) and (8).

Density functional theory (DFT) based quantum chemical calculations were carried out for compounds **5** and **8** to demonstrate their relative reactivity as an anti-inflammatory and antioxidant. The frontier molecular orbital pictures of **5** and **8** and their corresponding reactivity parameters are presented in Fig 11 and Table 6, respectively. The DFT/B3LYP approach was used in the current study to perform quantum chemical computations to optimize the selected structures. The DFT (B3LYP) method with 6-311G++(d,p) basis set was applied in this test. The electrostatic potential map, the optimized structure, and its HOMO and LUMO values are represented in Fig 12 HOMO energy expresses the ability of the compound to act as an electron donor. On the other hand, a site’s LUMO energy can act as an electron attractor. The electrostatic potential maps of the compounds show areas with electron localization throughout the molecules, with red and blue colors representing electron-rich (negative) and deficient (favorable) locations, respectively. Finally, Table 6 describes the DFT calculations that reveal favorable energetic parameters (E_HOMO_, E_LUMO_, ΔE, Ionization Energy IE, and Electron affinity EA) for the selected Compounds **5** and **8**.

**Table 6:** DFT-based reactivity parameters for compounds 5 and 8 derived using the Spartan 20 software package and the B3LYP/ 6-31 G (d,p) system.

S/N	E_HOMO_	E_LUMO_	ΔE	IE	EA
**5**	-6.02	0.72	5.3	6.02	-0.72
**8**	-9.06	-0.67	8.39	9.06	0.67

## 3. Experimental

### 3.1. Materials and methods

High-grade materials were used to synthesize the target compounds. Chemicals were obtained from Sigma-Aldrich (Taufkirchen, Germany0. Sigma-Aldrich Company provided solvents. The manufactured Mn_3_O_4_ nanoparticles were provided by the National Research Centre (NRC) by means of precipitation technique hemicals (ethyl cyanoacetate 99%, thiourea 99.5% and 4-cyanobenzaldehyde 99%, methyl iodide, chloroacetone, chloro-acetonitrile, acrylonitrile, ethyl chloroacetate, 2-amino benzothiazole, phenyl-hydrazine) were obtained from Sigma-Aldrich (Taufkirchen, Germany). Titanium (IV)isopropoxide (97%) was obtained from Merck Co. Solvents (ethanol 99.8%, pyridine, acetic anhydride, and acetic acid 99.7%) were provided by Sigma Aldrich Company. The National Research Center (…) provided TiO2 nanoparticles. The transmission Electron Microscope (TEM) sample was loaded on a carbon-coated Cu grid (200 mesh) and inspected at 200 kV utilizing JEM-2100 (JEOL, Tokyo, Japan)—electron microscopy unit- National Research Centre. The National Research Center screened the X-ray diffraction (XRD) pattern with the D8 Advance diffractometer (Bruker, Germany). Quantachrome Touch WinTM, model NOVA touch 4LX, measured specific surface area (SSA). The melting points were uncorrected and were measured by a digital Electrothermal IA 9100 Series apparatus Cole-Parmer, UK). A PerkinElmer CHN 2400 was utilized for C, H, and N analyses. IR spectra were obtained from 4000 to 400 cm^-1^ using FT-IR 460 PLUS (KBr disks). A Bruker 800 MHz NMR spectrometer was used for recording ^1^H and ^13^C-NMR spectra, the solvent utilized was DMSO-d_6_, and chemical shifts were obtained at KAUST and stated in δ (ppm). Cytotoxicity on Vero cell [HAV] was performed at the Pharmacology Department, Faculty of Veterinary Medicine, Cairo University, Cairo, Egypt.

### 3.2. Preparation of TiO_2_

Titania (TiO_2_) powders retain remarkable optical, dielectric, and catalytic properties, which point to industrial applications. The titanium (IV) isopropoxide hydrolysis process prepared the titanium dioxide nanoparticles. The preparatory solution was a combination of titanium (IV) isopropoxide, 98% (TTIP, ACROS organics, MW = 284.26), and isopropanol (CH_3_CHOHCH_3_). An appropriate volume of TTIP was dissolved in the proper amount of isopropanol (99.5%). A solution of distilled water with a pH value of 2 was used as a hydrolysis medium, and the pH was adjusted by adding nitric acid (HNO_3_). A pale yellow solution was produced when mixed under vigorous stirring. The resulting pale-yellow unclear solution was heated to 60-70°C for 18-20 h. The solution was kept under stirring until its volume decreased, and a refined suspension was produced. The yellowish-white suspension was dried at 100°C for 6h.

### 3.3. XRD

The formed phase of TiO_2_ was recognized from the X-ray diffraction (XRD) patterns inspected by the D8 Advance diffractometer (Bruker, Germany). The diffractogram was in terms of 2θ in the range of 10-80°. The JCPDS-International Center for Diffraction Data Cards was used as a reference for the documentation of the formed phases.

### 3.4. TEM

Particle size was confirmed by transmission electron microscopy (TEM; JEOL, Japan, JEM 2100, Electron Microscope, TEM-HR). First, particles were dispersed in ethanol, then a few milliliters of the solution were dropped onto a copper grid, and a TEM image was obtained.

### 3.5. Specific Surface Area (SSA)

SSA is a material property; it plays a significant role in the formation of nanoparticles, owing to the large surface-to-volume ratio and particle size reduction. Therefore, it has a precise significance in adsorption, heterogeneous catalysis, and surface reactions. Furthermore, the high surface area of the TiO_2_ nanoparticles enables the reaction/interaction between TiO_2_ and the interacting media, which mainly occurs on the surface or at the interface and is intensely influenced by the material’s surface area [37]. In the current study, Quantachrome TouchWinTM, model NOVA Touch 4LX measured the surface area of the prepared TiO2 at 169.339 (m2/gm).

### 3.6. Chemistry

#### 4-(4-cyanophenyl)-6-oxo-2-thioxohexahydropyrimidine-5-carbonitrile (1).

##### General procedure for (1)

###### Traditional method

A mixture of ethyl cyanoacetate (1.13 g, 10 mmol), thiourea (0.76 g, 10 mmol), and 4-cyanobenzaldehyde (1.31 g, 10 mmol) in 10 ml ethanol and a few drops of triethylamine was heated under reflux for 3 h. The solid product obtained after cooling was filtered off, washed with ethanol, and recrystallized from ethanol to yield yellow crystals (yield: 35%) m.p. 167-170°C.

###### Nanocatalytic method

To a mixture of ethyl cyanoacetate (1.13 g, 10 mmol), thiourea (0.76 g, 10 mmol), and 4-cyanobenzaldehyde (1.31 g, 10 mmol) in 10 ml ethanol, nano TiO_2_ (0.079 g, 1 mmol) was added. The reaction mixture was refluxed for 1 h, then filtered off on heat to remove the nanocatalyst. The reaction mixture was allowed to cool, and then the solid product obtained after cooling was filtered off, washed with ethanol, dried, and recrystallization from ethanol to afford yellow crystals (yield: 40%) m.p. 167-170 ^°^C.

#### 4-(4-cyanophenyl)-6-oxo-2-thioxohexahydropyrimidine-5-carbonitrile (1).

IR (KBr, ν, cm^-1^):3343-3521 cm^-1^ (2NH str), 3153- 2993 cm^-1^ (CH for aromatic and aliphatic), 2224 cm^-1^ (CN), 1721 cm^-1^(C = O), 1270 cm^-1^(C = S). ^1^H-NMR (DMSO-d_6_, 500 MHz): *δ = * 3.34 (*s*, 1*H,* methine-CH-CN), 4.34 (d,1H, CH(CN)- CH-Ar), 8.05,8.15 (*d*, d, 4*H*, CH Aromatic), and 8.49 ppm (s, 2*H*, 2NH). ^*13*^*C-NMR* (DMSO-d_6_, 500 MHz): *δ = *39.5, 62.7, 106.09, 114.5, 115.06, 118.14,131.001 133.01,135.5, 153.1, 161.2, 183.8 ppm*.MS:mle M*^* + *^*256.8, M*^* + 1*^*257.1, M-S atom 224.8. Anal. Calcd. for C*_*12*_*H*_*8*_*N*_*4*_*OS (256.28):C, 56.24; H, 3.15; N, 21.86; S, 12.51*

#### 4-(4-Cyanophenyl)-2-(methylthio)-6-oxo-1,4,5,6-tetrahydro pyrimidine-5-carbonitrile (2).

##### Traditional method

A solution of Compound **(1)** (2.56 g, 10 mmol) and methyl iodide (1.4 g, 10 mmol) in ethanolic sodium ethoxide (0.23 g, 10mmol/ 20 ml ethanol), the reaction mixture was refluxed for 3 h, then allowed to cool and poured into ice water. The solid product obtained after acidification with hydrochloric acid (6 ml, 30% soln.) was filtered off, washed with water, dried, and recrystallization from ethanol to afford a yellow powder (yield: 60%) m.p. 297-300^°^C.

##### Nanocatalytic Method

To a solution of Compound **(1)** (2.56 g, 10 mmol) and methyl iodide (1.4 g, 10 mmol) in ethanol (10 ml), nano TiO_2_ (0.079 g, 1 mmol) was added. The reaction mixture was refluxed for 2 h, then filtered off on heat to remove the nanocatalyst. The reaction mixture was allowed to cool, and then the solid product obtained after cooling was filtered off, washed with ethanol, dried, and recrystallization from ethanol to afford yellow crystals (yield: 72%) m.p. 297-300^°^C. IR (KBr, ν, cm^-1^): 3490 (NH, str), 3083-2858 (CH for aromatic and aliphatic), 2231 (CN), 1663 (C = O). ^1^H-NMR (DMSO-d_6_, 500 MHz): *δ = * 2.53(s, 3H, J = , CH_3_), 3.49 (*s*, 1*H, J = *,methine-CH-CN), 2.69 (*d*, 1*H, J = *, CH(CN)-CH-Ar), 8.05,8.09 (*d*,d, 4*H*, CH Aromatic), and 13.943 ppm (s, 1*H*, NH). ^*13*^*C-NMR* (DMSO-d_6_, 500 MHz): *δ = *13.39, 39.5, 40.41, 94.19, 113.88, 115.51, 118.25 (2CN), 129.53, 132.59, 139.61, 160.69, 165.72, 167.39 (C = O) ppm*. Anal. Calcd. fo*r*C*_*13*_*H*_*10*_ *N*_*4*_*OS (270.06): C, 57.76; H, 3.73; N, 20.73; S, 11.86*

#### 2-((Cyanomethyl)thio)-4-(4-cyanophenyl)-6-oxo-1,4,5,6-trtrahydropyrimidine-5-carbonitrile (3).

A solution of Compound **(1)** (0.256 g, 1 mmol) in ethanolic sodium ethoxide (0.0345 g, 1.5 mmol/ 5 ml ethanol) was stirred at room temperature for 1h, chloro-acetonitrile (0.09 g, 1.2 mmol) was added to the reaction mixture, followed by gentle heating for 5 h, then allowed to cool and poured into ice water. The solid product obtained after acidification with hydrochloric acid (6 ml, 30% soln.) was filtered off, washed with water, dried, and recrystallization from ethanol to afford a dark brown powder (yield: 80%) m.p. over 300^°^C. IR (KBr, ν, cm^-1^): 3450 (NH, str),2235, 2204 (2CN), 1642 (C = O). ^1^H-NMR (DMSO-d_6_, 500 MHz): δ *= * 3.61 (*s*, 1H, J = , (methine-CH-CN), 2.49 (*d*, 1H, J = , CH(CN)-CH (Ar), 4.16(s,2H, -S-CH_2_-CN), 7.914, 7.931 (*d*, 4H, CH Aromatic), and 8.06 (s, 1H, NH) ppm. Anal. Calcd. for C_14_H_9_ N_5_ OS (295.32): C, 56.94; H, 3.07; N, 23.71; S, 10.86.

#### 4-(4-Cyanophenyl)-6-oxo-2-((2-oxo-propyl)thio)-1,4,5,6-tetrahydro-pyrimidine-5-carbonitrile (4).

A mixture of Compound **(1)** (0.256 g, 1 mmol) and chloroacetone (0.11 g, 1.2 mmol) in ethanol (5 ml) and a few drops of triethylamine was allowed to reflux for 4 h; after that, the reaction mixture was cooled. The precipitated solid product was filtered off, washed with ethanol, dried, and recrystallized from ethanol to yield a pale brown powder (yield: 65%) m.p. over 300^°^C. IR (KBr, ν, cm^-1^): 3427 (NH, str), 3065-2971(CH for aromatic and aliphatic), 2228 (CN), 1738, 1664 (2 C = O). ^1^H-NMR (DMSO-d_6_, 500 MHz): *δ = * 2.2 (s, 3H, J = , CO- CH_3_), 4.16 (s, 2H, J = , S-CH_2_-CO), 3.33(d,1H, CH(CN)-CH(Ar), 5.297 (s, 1H, methine-CH-CN, 8.087, 8.100 (d, 4H, Aromatic) ppm. Anal. Calcd. for C_15_H_12_N_4_ O _2_S (312.35): C, 57.68; H, 3.87; N, 17.94; S, 10.26

#### 4-Amino-8-(4-cyanophenyl)-6-oxo-7,8-dihydro-2H,6H-pyrimido[2,1-b][1,3]thiazine-7-carbonitrile (5).

##### Traditional method

To a solution of Compound **(1)** (2.56 g, 10mmol) in (20 ml) pyridine, acrylonitrile (0.53 g, 10mmol) was added, and the reaction mixture was refluxed for 5h. After cooling at room temperature, the reaction mixture was poured into ice water. The solid product was obtained after acidification with hydrochloric acid (6 ml, 30% soln.), filtered off, washed with water, dried, and recrystallized from ethanol to afford a yellow powder (yield: 45%) m.p. 240-245°C. F

##### Nanocatalytic method

To a solution of Compound **(1)** (0.256 g, 1 mmol) and acrylonitrile (0.053 g, 1 mmol) in ethanol (5 ml), nano TiO_2_ (0.008 g, 10 mmol) was added. The reaction mixture was refluxed for 3 h, then filtered off on heat to remove the nanocatalyst. The reaction mixture was allowed to cool, and then the solid product obtained after cooling was filtered off, washed with ethanol, dried, and recrystallization from ethanol to yield yellow crystals (yield: 57%) m.p. 240- 245^°^.(yield: 45-70%) m.p. 240-245°C. IR (KBr, ν, cm^-1^): 3157-3491(NH_2_, str), 3085-2870(CH for aromatic and aliphatic), 2230 (CN), 1678 (C = O). ^1^H-NMR (DMSO-d_6_, 500 MHz): *δ = 2.49(d,1H, CH(CN)-CH(Ar),* 3.1 (s,1H, methine-CH(CN)),3.64 (*d*, 2*H,* S-CH_2_-CH) 4.3(t,1H, S-CH_2_CH), 7.85,7.86 (*d*, 4*H*, Aromatic), and 8.9 (s, 2*H*, NH_2_) ppm*. Anal. Calcd. fo*r*C*_*15*_*H*_*11*_*N*_*5*_*OS (309.35): C, 58.24; H, 3.58; N, 22.64; S, 10.36*

#### 7-(4-Cyanophenyl)-3,5-dioxo-2,3,6,7-tetrahydro-5H-thiazolo[3,2-a]pyrimidine-6-carbonitrile (6).

A mixture of compound **1**(0.256 g, 1 mmol) and ethyl chloroacetate (0.1 g, 1.2 mmol) in ethanol (5 ml) and a few drops of triethylamine was allowed to reflux for 6 h; after that, the reaction mixture was cooled.Theprecipitated solid product was filtered off, washed with ethanol, dried, and recrystallized from ethanol to afford a pale yellow powder (yield: 55%) m.p. over 265-270^°^C. IR (KBr, ν, cm^-1^): 3131-2966(CH for aromatic and aliphatic), 2229 (CN), 1675,1615 (2 C = O). ^1^H-NMR (DMSO-d_6_, 500 MHz): *δ = 2.49(d,1H,CH(CN)-CH(Ar)* 3.36 (*s*, 1*H, J = *, methine-CH(CN)),3.99(s,2H, CH_2_thiazole ring) 7.739,7.951 (*d*,d, 4*H*, CH Aromatic) ppm*. Anal. Calcd. fo*r C_14_H_8_N_4_O_2_S *(296.30):C, 56.75; H, 2.72; N, 18.91; S, 10.82.*

#### (E)-2-benzylidene-7-(4-cyanophenyl)-3,5-dioxo-2,3,6,7-tetrahydro-5H-thiazolo[3,2-a]pyrimidine-6-carbonitrile (7).

A mixture of Compound **1** (0.256 g, 1mmol), chloroacetic acid (0.113 g, 1.2 mmol), benzaldehyde (0.127 g, 1.2 mmol), and anhydrous sodium acetate (0.328 g, 4 mmol) in a mixture of acetic acid and acetic anhydride (1:1) was heated under reflux for 7 h. The formed solid was collected and washed with acetic acid and ethanol several times, dried, and recrystallized from ethanol to give a greenish-yellow powder (yield: 60%) m.p. 270-275°C. IR (KBr, ν, cm^-1^): 2228 (CN), 1761,1694 (2 C = O). ^1^H-NMR (DMSO-d_6_, 500 MHz): δ = 2.49(d,1H, CH(CN)-CH(Ar) 3.35 (s, 1H, J = , methine-CH(CN)), 4.5(s,1H, C = CH benzylidene), 7.771, 7.787(m, 9H, aromatic) ppm. Anal. Calcd. for C_21_H_12_N_4_O_2_S (384.41):C, 65.61; H, 3.15; N, 14.57; S, 8.34.

#### 2-(Benzo[d]thiazol-2-ylamino)-4-(4-cyanophenyl)-6-oxo-1,4,5,6-tetrahydro pyrimidine-5-carbonitrile (8).

A mixture of Compound **(2)** (0.27 g, 1 mmol) and 2-aminobenzothiazole (0.15 g, 1 mmol) in ethanol (10 ml) and a few drops of triethylamine was refluxed for 5 h, then cooled at room temperature. After that, the reaction mixture was poured into ice water. The precipitated solid product was filtered off, washed with ethanol, dried, and recrystallized from ethanol to afford a pale brown powder (yield: 70%) m.p. 285-290^°^C. IR (KBr, ν, cm^-1^): 3448,3422 (2 NH, str), 3099-2926 (CH for aromatic and aliphatic), 2230 (CN), 1676 (C = O). ^1^H-NMR (DMSO-d_6_, 500 MHz): δ =  3.06 (d, 1H, J = , CH(CN)-CH (Ar)), 3.48 (s, 1H, J = , methine-CH(CN)), 9.92, 11,95 (s, 2H,2 NH, str) 7.85- 7.47 (m, 8H, CH Aromatic) ppm. Anal. Calcd. forC19H12N6OS (372.08): C, 61.28; H, 3.25; N, 22.57; S, 8.61.

#### 4-(4-Cyanophenyl)-6-oxo-2-(2-phenylhydrazine)-1,4,5,6-tetrahydro pyrimidine-5-carbonitrile (9).

A mixture of Compound **(2)** (0.27 g, 1 mmol) and phenyl-hydrazine (0.108 g, 1 mmol) in ethanol (10 ml) and a few drops of triethylamine was refluxed for 4 h, then cooled at room temperature. After that, the reaction mixture was poured into ice water. The precipitated solid product was filtered off, washed with ethanol, dried, and recrystallized from ethanol to afford a pale brown powder (yield: 55%) m.p. over 300^°^C. IR (KBr, ν, cm^-1^): 3449 (NH, str), 2224 (CN), 1669 (C = O). ^1^H-NMR (DMSO-d_6_, 500 MHz): *δ = * 2.62 (*d*, 1H, J = , CH(CN)-CH (Ar)), 3.37 (s, 1H, J = , methine-CH(CN)), 8.205-8.189-(d, d,2H,2 NH, str) 8.002(s, 1H, NH, str) 7.693- 7.624 (m, 9H, CH aromatic) ppm. Anal. Calcd. for C_18_H_14_N_6_O (330.35): C, 65.44; H, 4.27; N, 25.44

### 3.7. Biology

#### 3.7.1 Evaluation of antioxidant activity by DPPH radical scavenging method.

Different compounds’ free radical scavenging activity was measured by 1, 1- diphenyl-2-picryl hydroxyl (DPPH). In brief, a 0.1 mM solution of DPPH in ethanol was prepared. This solution (1 ml) was added to 3 ml. of the different compounds in ethanol at various concentrations (3.9, 7.8, 15.62, 31.25, 62.5, 125, 250, 500, 1000 μg/ml). Here, only those compounds solubilized in ethanol and their various concentrations were prepared by dilution [38]. The mixture was shaken vigorously and allowed to stand at room temperature for 30 minutes. Then, absorbance was measured at 517 nm by using a spectrophotometer (UV-VIS Milton Roy). The reference standard compound used was ascorbic acid, and the experiment was done in triplicate. The IC 50 value of the sample, which is the concentration of sample required to inhibit 50% of the DPPH free radical, was calculated using the Log dose inhibition curve. The lower absorbance of the reaction mixture indicates higher free radical activity. The percent DPPH scavenging effect was calculated using the following equation: DPPH scavenging effect (%), or percent inhibition =  A0 - A 1/ A0 ×  100. A0 is the absorbance of the control reaction, and A1 is the absorbance in the presence of a test or standard sample [39].

#### 3.7.2 Hemolytic assay.

The hemolytic assay was performed using the method reported in [37,40,41]. Freshly collected human red blood cells were washed thrice with 150 mM NaCl (2500 rpm for 10 minutes). The plasma was removed, and the cells were suspended in phosphate buffer saline (pH 7.4) for 2% RBC concentration. Double-folded dilution concentrations (1000, 800, 600, 400, 200, 100, 50 μg/ml) of the compound were mixed with 2% L of RBC solutions, and the final reaction mixture volume was measured up to 1 ml by adding PBS. The reaction mixture was then placed in the water bath for 1 hour at 37°C. After incubation, the reaction mixture was centrifuged at 2500 rpm for 15 minutes. The supernatant was collected, and the optical density was measured at 541 nm, keeping the phosphate buffer saline blank. Deionized water was used as a positive control. The experiment was carried out in triplicate, and the mean ±  SD was calculated.


$$\begin{array}{l} Percentageofhemolysis=\left(Absorbanceofsample-Absorbanceofblank\right)\times \\ 100/Absorbanceofpositivecontrol \end{array}$$

### 3.8 In-silico studies

#### 3.8.1 Molecular docking.

The docking analyses were carried out and characterized by the Molecular Environment (MOE), ChemDraw 16 compound preparation, Chem3D structures, and Chem 3D 16 (Molecular Modeling and Analysis; Cambridge Soft Corporation) software. To dock the co-crystal structure of the COX-2 complex with SC-558, a selective COX-2 blocker was acquired from the PDB (protein data bank) **PDB: 1CX2** and co-crystallized with ascorbic acid as a reference ligand isolated from cytochrome *c* peroxidase enzyme **PDB: 2X08**. After the crystal structure was downloaded from the PDB www.rcsb.org, the water molecules, co-ligand, and metal ions were removed [42–44–].

The final form was obtained after 3D protonation and the correction process. The MOE site finder generated the active binding sites to create the dummy sites as the binding pocket. The default docking parameters were triangle matches for replacing the molecule and London dG for rescoring the docking scores. The DFT-optimized structures of the compounds were used to generate the five best binding poses with flexible molecule rotation. The hydrogen bonds formed between the elastase and the investigated compound were used to rank the binding affinity and were presented as the free binding energy (S, kcal/mol). The higher negative values of the docking scores were presented along with 2D and 3D structures [45,46].

#### 3.8.2 Pharmacokinetics.

##### LabMol and ProTox-II.

Computational toxicity estimations are faster than determining toxic doses in animals and also help reduce the number of animal experiments. Toxic doses are often given as LD50 in mg/kg body weight. The LD50 is the median lethal dose at which 50% of test subjects die upon exposure to a compound. Toxicity classes are defined according to the globally harmonized system of classification and labeling of chemicals (GHS) [47].

LD50 values are given in [mg/kg]:

1- Class I: fatal if swallowed (LD50 ≤  5).
2- Class II: fatal if swallowed (5 < LD50 ≤  50).
3- Class III: toxic if swallowed (50 < LD50 ≤  300).
4- Class IV: harmful if swallowed (300 < LD50 ≤  2000).
5- Class V: may be harmful if swallowed (2000 < LD50 ≤  5000)
6- Class VI: non-toxic (LD50 >  5000)

The ProTox-II software predicts different toxicity endpoints, such as acute toxicity, hepatotoxicity, carcinogenicity, and mutagenicity [48]. The Pred-hERG (human Ether-a-go-go-Related Gene) software was used to assess cardiotoxicity. The software depends on statistically significant and externally predictive quantitative structure-activity relationship (QSAR) models of hERG blockage closely associated with severe and potentially fatal cardiac dysrhythmia. The SDF (structure data file) and SMILES (simplified molecular-input line-entry system) strings were used throughout the generation process [48].

#### Molinspiration.

##### Bioavailability radar

This is a tool for rapid appraisal of the drug-likeness of a molecule. Six physicochemical properties (lipophilicity, size, polarity, solubility, flexibility, and saturation) were considered. On each axis, a physicochemical range was determined by descriptors as previously explained [49] and illustrated as a pink area in which the radar plot of the compound must fall in its entirety to be considered drug-like. Pro-Tox-II carried this out.

##### Physicochemical properties

Physiochemical properties include simple molecular and physicochemical descriptors such as molecular weight (MW), number of specific atom types, fraction Csp3 (carbon bond saturation as defined by fraction sp3), which measure the complexity of the molecule, number of particular bond types, and molecular refractivity (MR). Also, the topological polar surface area (TPSA) was used to calculate the polar surface area (PSA), which quickly estimates some ADME properties, especially concerning the ability to pass through biological barriers like the blood-brain barrier [50].

##### Lipophilicity

Lipophilicity was described by calculating the partition coefficient between *n*-octanol and water (Log *P*o/w). Then, Molinspiration gave access to five free predictors, iLOGP, XLOGP3, WLOGP, MLOGP, and SILICO—IT, to generate the consensus log *P*o/w, which is the mean of the predicted values [51].

##### Water solubility

Molinspiration predicts water solubility [52]. The output is the Log S values, the decimal Log p of the molar solubility in water. In addition, the water solubility was provided in mg/ml and mol/l, as were the qualitative solubility classes.

##### Pharmacokinetics

Molinspiration uses specialized models to evaluate the ADME behaviors of the test compound. Lipinski’s rule of five (5) by Christopher A. Lipinski 1997 is a rule of thumb for assessing drug-likeness and determining if an inhibitor with specific biological and pharmacological properties would be an orally active drug in the human body. The rule states that a molecule can be orally absorbed/active if two (2) or more of these thresholds obtain: molecular weight (Mw) of molecule <  500, octanol/water partition coefficient (ilog P) _ 5, number of hydrogen bond acceptors (nHBA) _ 10, number of hydrogen bond donors (nHBD) _ 5, and topological polar surface area (TPSA) <  40 Å2), must not be violated. The first model predicts passive gastrointestinal absorption and penetration of the blood-brain barrier (BBB) [53]. The second model predicts being substrate or non-substrate of the permeability glycoprotein (P-GP), essential to evaluate active efflux through membranes, e.g., from the gastrointestinal wall to the lumen or brain [41]. The third model predicts the interaction of compounds with the cytochrome P450 (CYP) major isoenzymes (CYP1A2, CYP2C19, CYP2C9, CYP2D6, CYP3A4), which is an essential contributor to drug elimination through metabolic biotransformation. Also, inhibition of these isoenzymes is a cause of drug interactions [37], leading to toxic or other adverse effects. The fourth model predicts the skin permeability coefficient (*K*p), which linearly correlates with molecular size and lipophilicity [54]—the more negative the log *Kp*(cm/s), the less skin permeation.

#### 3.8.3 DFT.

The Spartan ‘14 program was used to perform quantum chemistry calculations using the DFT method. In addition, spartan ‘20 was used to display all the data files. The density functional theory (DFT) at 6-311G^++^(d,p) basis set/B3LYP approach was utilized to optimize the organic chemical structure of the compounds under investigation, and Chem3D 16.0 software was used to create the original chemical structure [53,54].

## Conclusion

A cost-effective and eco-friendly protocol has been developed by using the Biginelli synthesis of 4-cyanobenzaldehyde, ethyl cyanoacetate, and thiourea in the presence of TiO_2_ nanoparticles to yield 4-(4-cyanophenyl)-6-oxo-2-thioxohexahydropyrimidine-5-carbonitrile (1). This multi-component system is safe and non-toxic. Compound **(1)** was reacted with different reagents, as listed above, to produce new compounds. These compounds were then studied for inflammatory and antioxidant activity. Compounds **(5)** and **(8)** showed great activity with IC_50._ The in-silico studies include molecular docking using MOE and pharmacokinetics using Toxoradar, SAR, and DFT studies.

## Supporting information

S1 Fig 4-(4-cyanophenyl)-6-oxo-2-thioxohexahydropyrimidine-5-carbonitrile (1): (PDF)

S2 Fig 4-(4-Cyanophenyl)-2-(methylthio)-6-oxo-1,4,5,6-tetrahydropyrimidine-5-carbonitrile (2): (PDF)

S3 Fig 2-((cyanomethyl)thio)-4-(4-cyanophenyl)-6-oxo-1,4,5,6-tetrahydropyrimidine-5-carbonitrile (3) (PDF)

S4 Fig 4-(4-cyanophenyl)-6-oxo-2-((2-oxopropyl)thio)-1,4,5,6-tetrahydropyrimidine-5-carbonitrile (4): (PDF)

S5 Fig 4-Amino-8-(4-cyanophenyl)-6-oxo-7,8-dihydro-2H,6H-pyrimido[2,1-b][1,3]thiazine-7-carbonitrile (5) (PDF)

S6 Fig 7-(4-Cyanophenyl)-3,5-dioxo-2,3,6,7-tetrahydro-5H-thiazolo[3,2-a]pyrimidine-6-carbonitrile (6) (PDF)

S7 Fig (E)-2-benzylidene-7-(4-cyanophenyl)-3,5-dioxo-2,3,6,7-tetrahydro-5H-thiazolo[3,2-a]pyrimidine-6-carbonitrile (7) (PDF)

S8 Fig 2-(Benzo[d]thiazol-2-ylamino)-4-(4-cyanophenyl)-6-oxo-1,4,5,6-tetrahydropyrimidine-5-carbonitrile (8) (PDF)

S9 Fig 4-(4-Cyanophenyl)-6-oxo-2-(2-phenylhydrazineyl)-1,4,5,6-tetrahydropyrimidine-5-carbonitrile (9) (PDF)

S1 Graphical Abstract New pyrimidine candidates,  antioxidant activity, anti-inflammatory effects, and insilico-studies. (PPTX)
