# Supplementary material for: Innovation of eco-friendly TiO2 nano catalyst for new pyrimidine carbonitiriles candidates, assessed for significant antioxidant activity, anti-inflammatory effects, and by insilico studies
Source: PLoS One. 2025 May 29;20(5):e0313959. doi: 10.1371/journal.pone.0313959 (PMC12121771; doi:10.1371/journal.pone.0313959)

Spectral Analysis

S Fig 1: 4-(4-cyanophenyl)-6-oxo-2-thioxohexahydropyrimidine-5-carbonitrile (1):

IR Spectrum of (1):

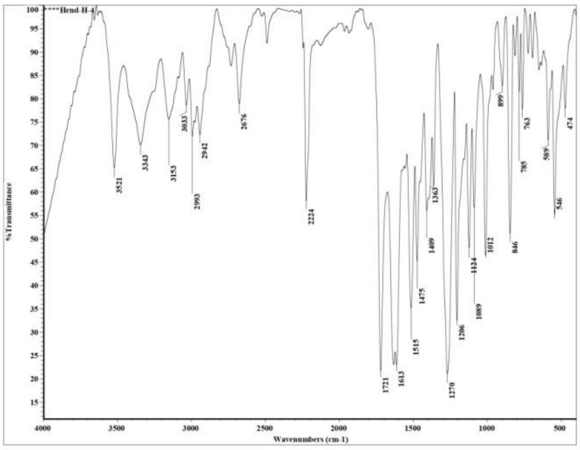

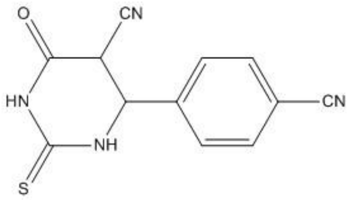

1

^{1}H-NMR Spectrum of (1):

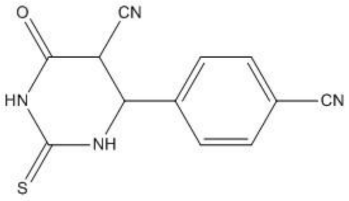

1

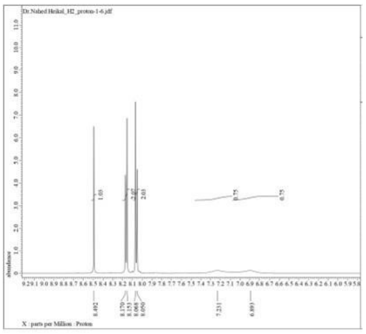

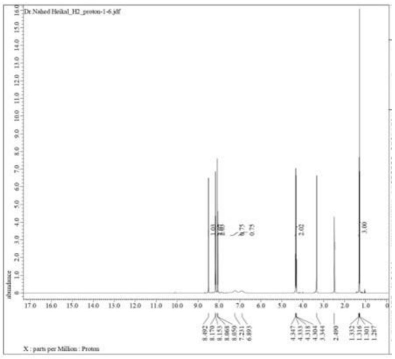

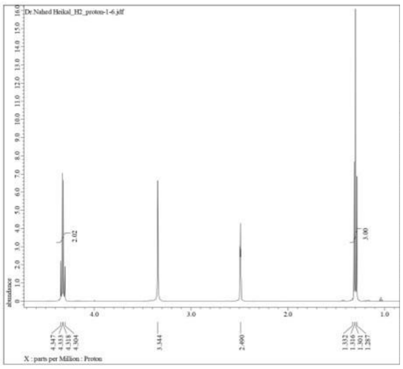

-

^{13}C-Spectrum of (1)

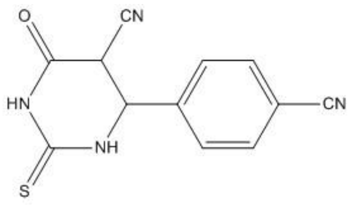

1

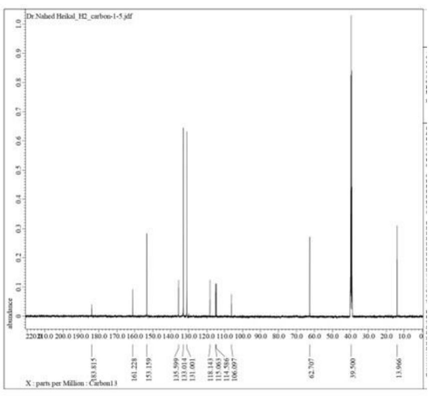

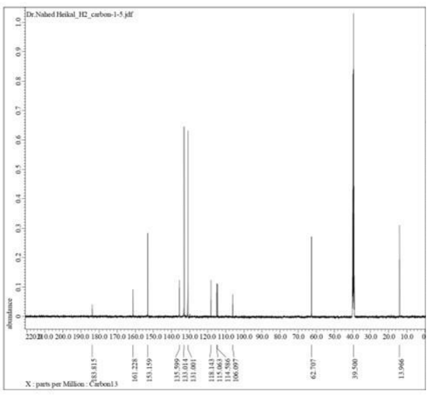

Supplement: S1 Fig — (PDF) [file pone.0313959.s001.pdf]
