# Supplementary material for: Innovation of eco-friendly TiO2 nano catalyst for new pyrimidine carbonitiriles candidates, assessed for significant antioxidant activity, anti-inflammatory effects, and by insilico studies
Source: PLoS One. 2025 May 29;20(5):e0313959. doi: 10.1371/journal.pone.0313959 (PMC12121771; doi:10.1371/journal.pone.0313959)

S Fig 2 : 4-(4-Cyanophenyl)-2-(methylthio)-6-oxo-1,4,5,6-tetrahydropyrimidine-5-carbonitrile (2):

IR Spectrum of (2):

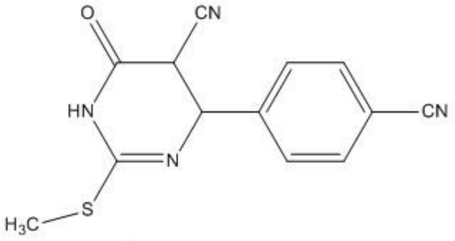

2

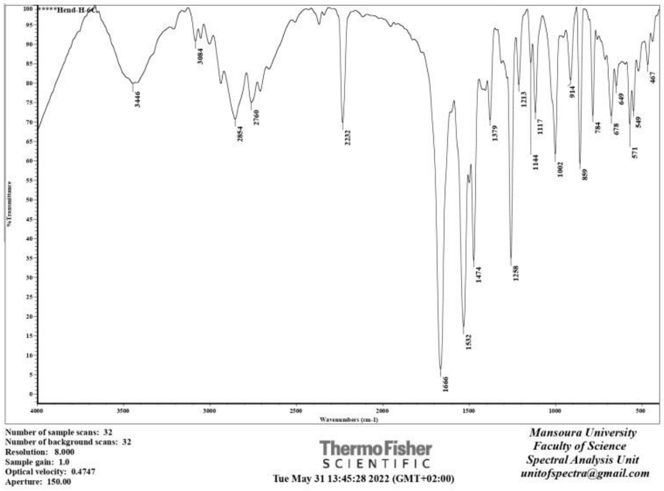

^{1}H-NMR Spectrum of (2):

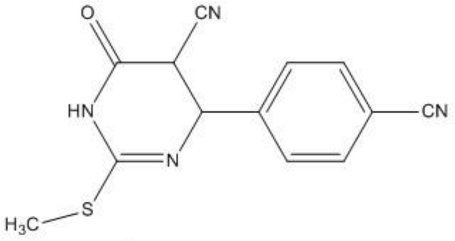

2

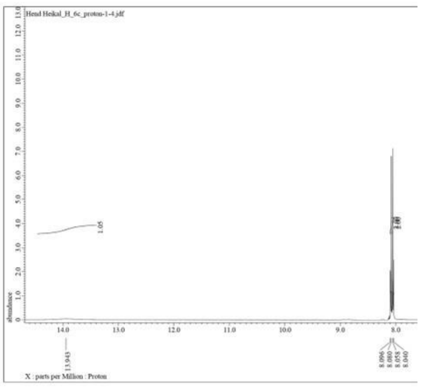

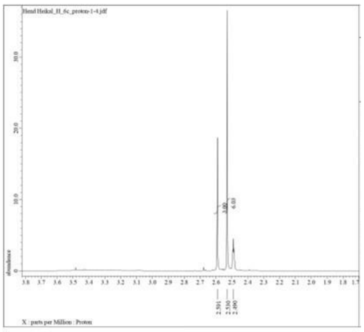

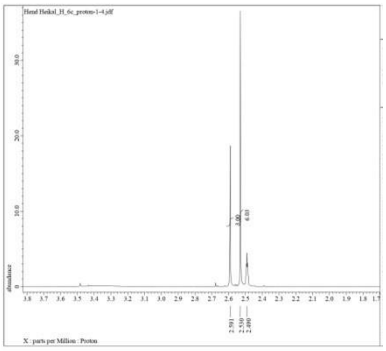

^{13}C-Spectrum of (2):

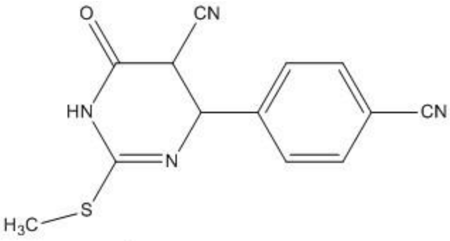

2

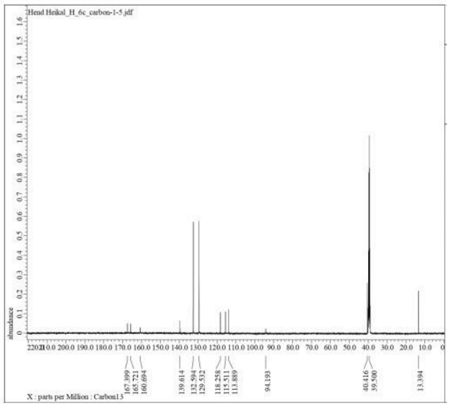

Supplement: S2 Fig — (PDF) [file pone.0313959.s002.pdf]
