# Supplementary material for: Innovation of eco-friendly TiO2 nano catalyst for new pyrimidine carbonitiriles candidates, assessed for significant antioxidant activity, anti-inflammatory effects, and by insilico studies
Source: PLoS One. 2025 May 29;20(5):e0313959. doi: 10.1371/journal.pone.0313959 (PMC12121771; doi:10.1371/journal.pone.0313959)
Supplement: S4 Fig — (PDF) [file pone.0313959.s004.pdf]

S Fig 4 :4-(4-cyanophenyl)-6-oxo-2-((2-oxopropyl)thio)-1,4,5,6-tetrahydropyrimidine-5-carbonitrile **(4)**:

IR Spectrum of (4):

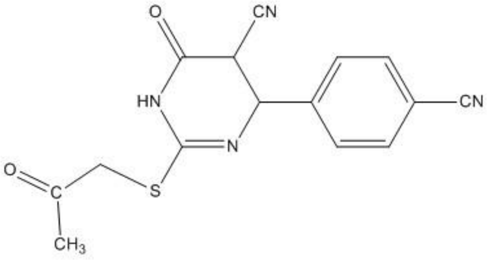

4

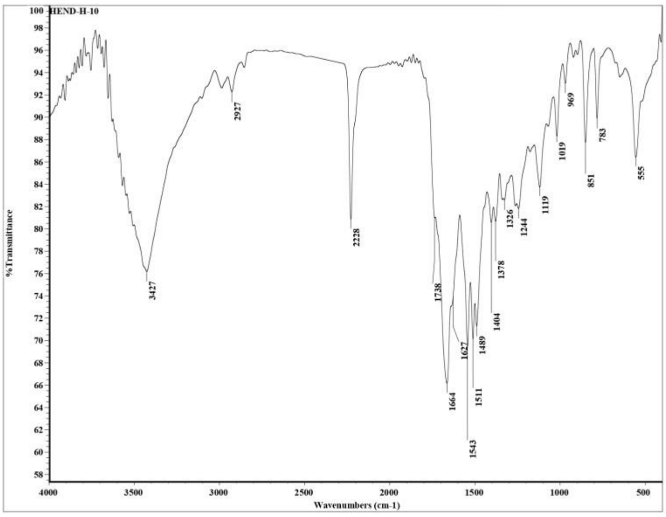

^{1}H-NMR Spectrum of (4):

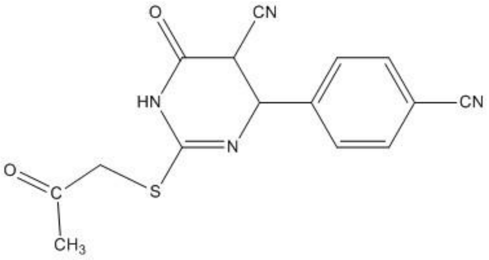

4

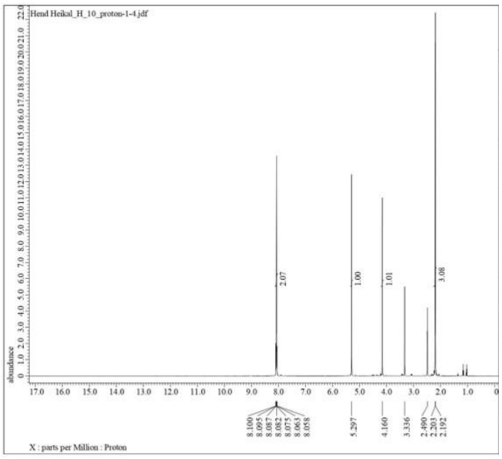
Hend Heikal_H_10_proton-1-4.jdf
abundance
2.07
1.00
1.01
3.08
8.100
8.095
8.087
8.082
8.075
8.063
8.058
5.297
4.160
3.336
2.490
2.203
2.192
X : parts per Million : Proton

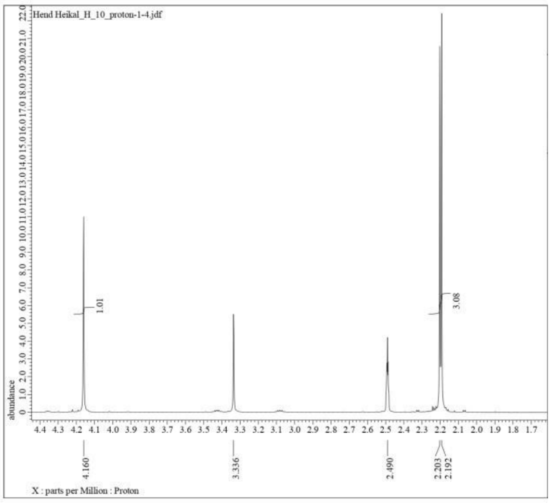
Hend Heikal_H_10_proton-1-4.jdf
abundance
1.01
3.08
4.160
3.336
2.490
2.203
2.192
X : parts per Million : Proton

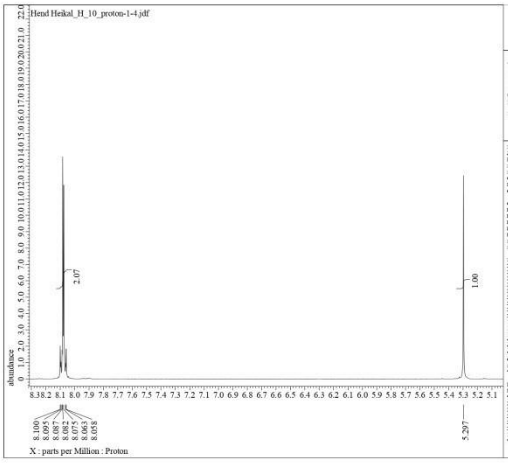
Hend Heikal_H_10_proton-1-4.jdf
abundance
2.07
1.00
8.100
8.095
8.087
8.082
8.075
8.063
8.058
5.297
X : parts per Million : Proton
