# Supplementary material for: Innovation of eco-friendly TiO2 nano catalyst for new pyrimidine carbonitiriles candidates, assessed for significant antioxidant activity, anti-inflammatory effects, and by insilico studies
Source: PLoS One. 2025 May 29;20(5):e0313959. doi: 10.1371/journal.pone.0313959 (PMC12121771; doi:10.1371/journal.pone.0313959)

S Fig 5: 4-Amino-8-(4-cyanophenyl)-6-oxo-7,8-dihydro-2H,6H-pyrimido[2,1-b][1,3]thiazine-7-carbonitrile (5):

IR Spectrum of (5):

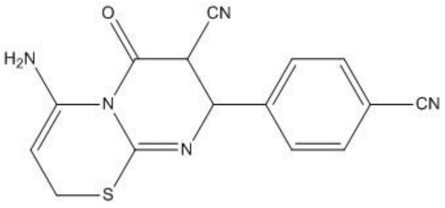

5

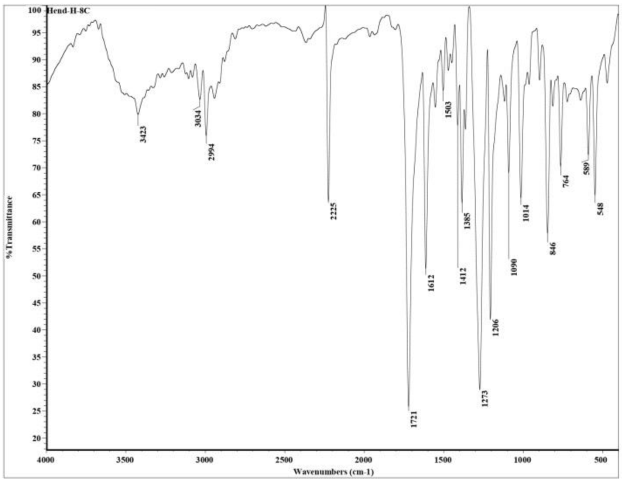

^{1}H-NMR Spectrum of (5):

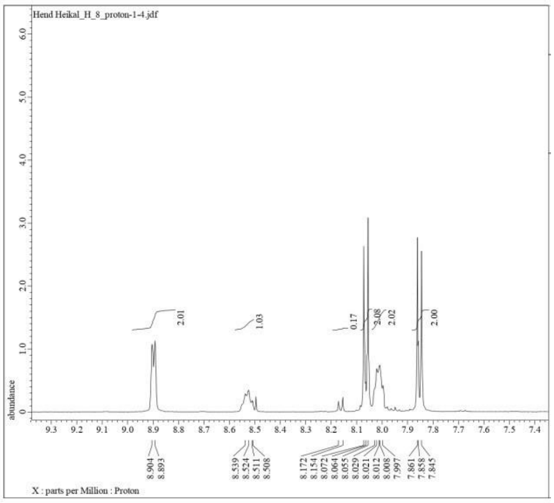

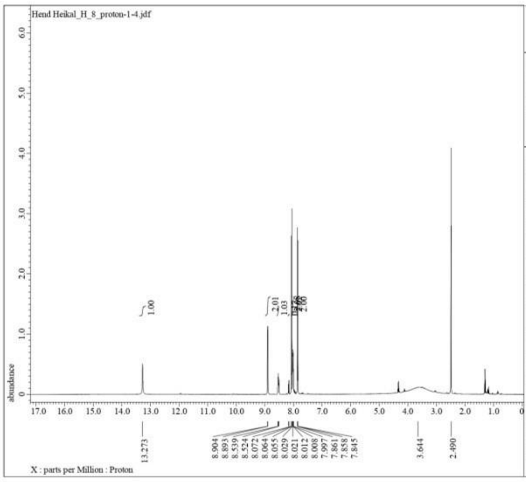

Supplement: S5 Fig — (PDF) [file pone.0313959.s005.pdf]
