# Supplementary material for: Innovation of eco-friendly TiO2 nano catalyst for new pyrimidine carbonitiriles candidates, assessed for significant antioxidant activity, anti-inflammatory effects, and by insilico studies
Source: PLoS One. 2025 May 29;20(5):e0313959. doi: 10.1371/journal.pone.0313959 (PMC12121771; doi:10.1371/journal.pone.0313959)
Supplement: S6 Fig — (PDF) [file pone.0313959.s006.pdf]

S Fig 6: 7-(4-Cyanophenyl)-3,5-dioxo-2,3,6,7-tetrahydro-5H-thiazolo[3,2-a]pyrimidine-6-carbonitrile (6):

IR Spectrum of (6):

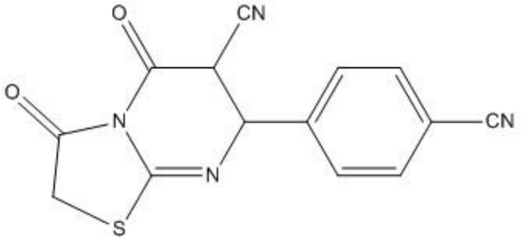

6

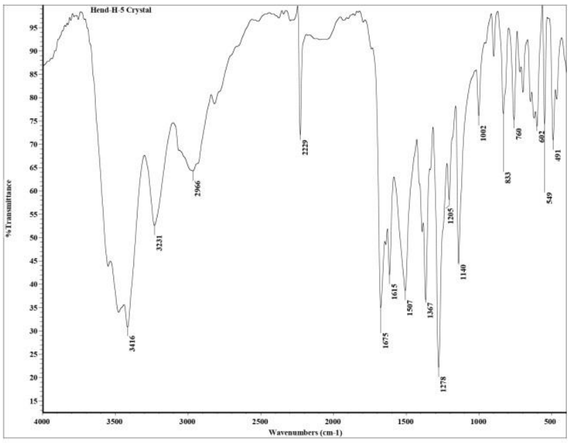

^{1}H-NMR Spectrum of (6):

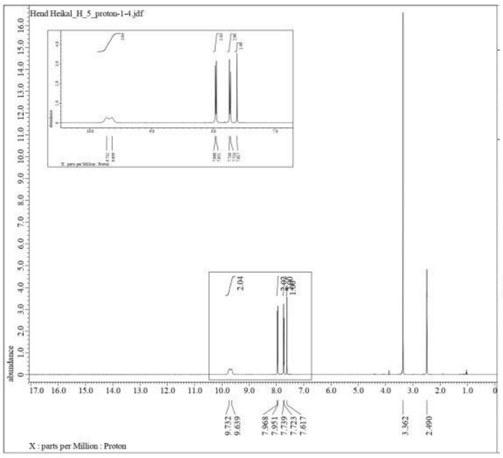

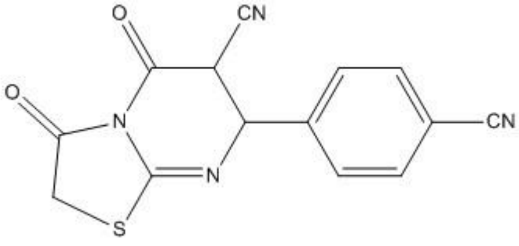

6
