# Supplementary material for: Innovation of eco-friendly TiO2 nano catalyst for new pyrimidine carbonitiriles candidates, assessed for significant antioxidant activity, anti-inflammatory effects, and by insilico studies
Source: PLoS One. 2025 May 29;20(5):e0313959. doi: 10.1371/journal.pone.0313959 (PMC12121771; doi:10.1371/journal.pone.0313959)
Supplement: S7 Fig — (PDF) [file pone.0313959.s007.pdf]

S Fig 7: (E)-2-benzylidene-7-(4-cyanophenyl)-3,5-dioxo-2,3,6,7-tetrahydro-5H-thiazolo[3,2-a]pyrimidine-6-carbonitrile (7):

IR Spectrum of (7):

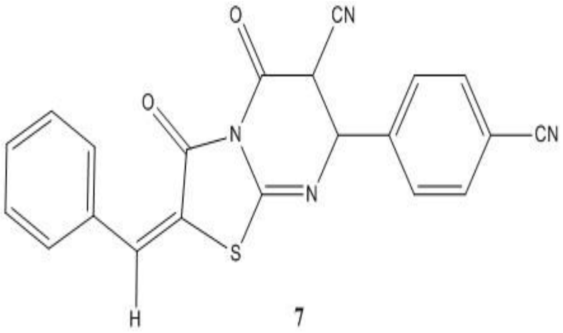

7

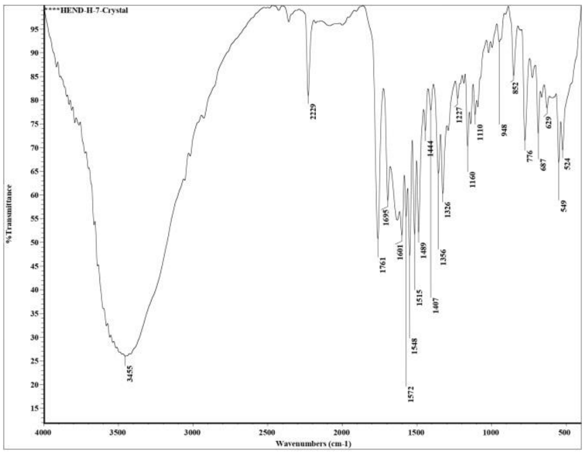

^{1}H-NMR Spectrum of (7):

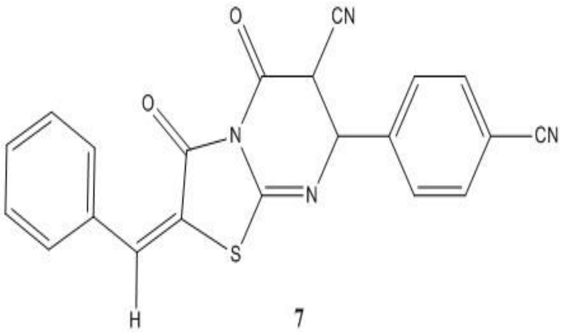

7

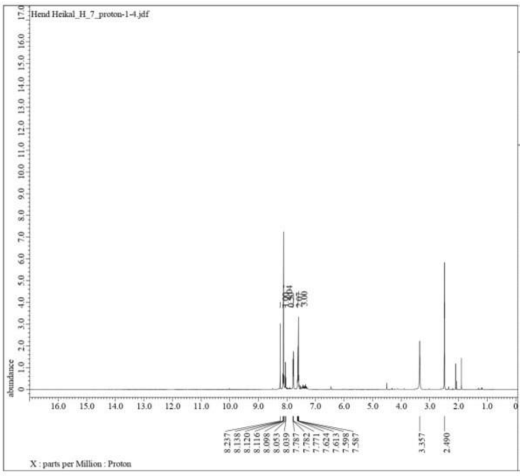

Hend Heikal_H_7_proton-1-4.jdf
abundance
8.237
8.138
8.120
8.116
8.098
8.053
8.039
7.787
7.782
7.771
7.624
7.613
7.598
7.587
3.357
2.490
X : parts per Million : Proton
