# Supplementary material for: Innovation of eco-friendly TiO2 nano catalyst for new pyrimidine carbonitiriles candidates, assessed for significant antioxidant activity, anti-inflammatory effects, and by insilico studies
Source: PLoS One. 2025 May 29;20(5):e0313959. doi: 10.1371/journal.pone.0313959 (PMC12121771; doi:10.1371/journal.pone.0313959)
Supplement: S8 Fig — (PDF) [file pone.0313959.s008.pdf]

S Fig 8; 2-(Benzo[d]thiazol-2-ylamino)-4-(4-cyanophenyl)-6-oxo-1,4,5,6-tetrahydropyrimidine-5-carbonitrile (8):

IR Spectrum of (8):

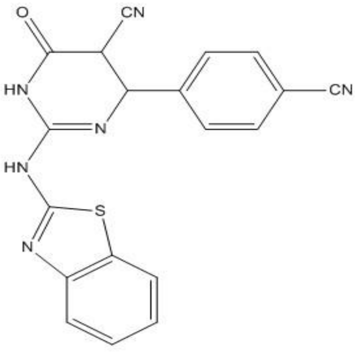

8

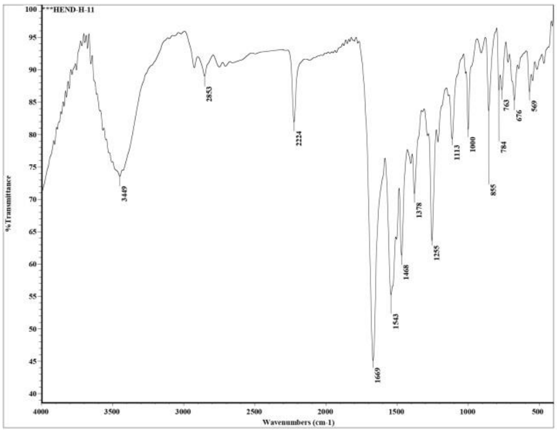

^{1}H-NMR Spectrum of (8):

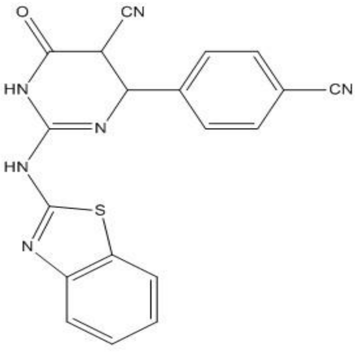

8

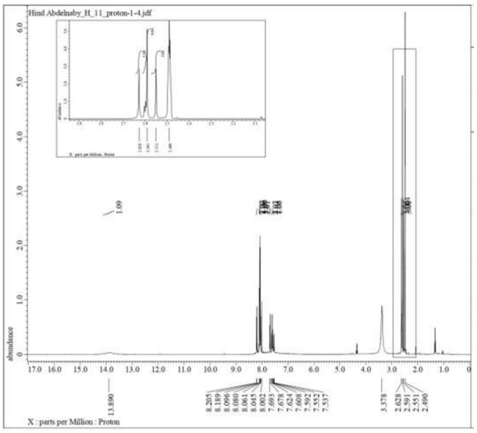
Hind Abdelnaby_H_11_proton-1-4.jdf
abundance
X : parts per Million : Proton
13.890
8.205
8.189
8.096
8.080
8.061
8.045
8.002
7.693
7.678
7.624
7.608
7.592
7.552
7.537
3.378
2.628
2.591
2.551
2.490
1.09

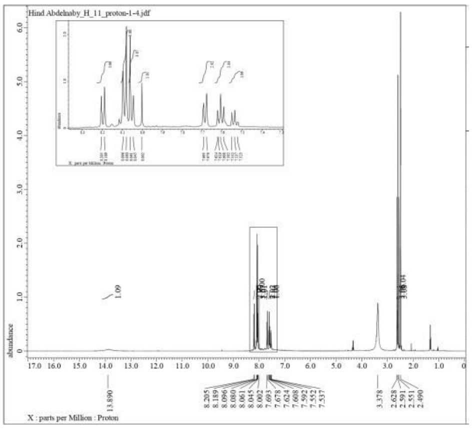
Hind Abdelnaby_H_11_proton-1-4.jdf
abundance
X : parts per Million : Proton
13.890
8.205
8.189
8.096
8.080
8.061
8.045
8.002
7.693
7.678
7.624
7.608
7.592
7.552
7.537
3.378
2.628
2.591
2.551
2.490
1.09
