# Supplementary material for: Innovation of eco-friendly TiO2 nano catalyst for new pyrimidine carbonitiriles candidates, assessed for significant antioxidant activity, anti-inflammatory effects, and by insilico studies
Source: PLoS One. 2025 May 29;20(5):e0313959. doi: 10.1371/journal.pone.0313959 (PMC12121771; doi:10.1371/journal.pone.0313959)
Supplement: S9 Fig — (PDF) [file pone.0313959.s009.pdf]

S Fig 9 : 4-(4-Cyanophenyl)-6-oxo-2-(2-phenylhydrazineyl)-1,4,5,6-tetrahydropyrimidine-5-carbonitrile (9):

IR Spectrum of (9):

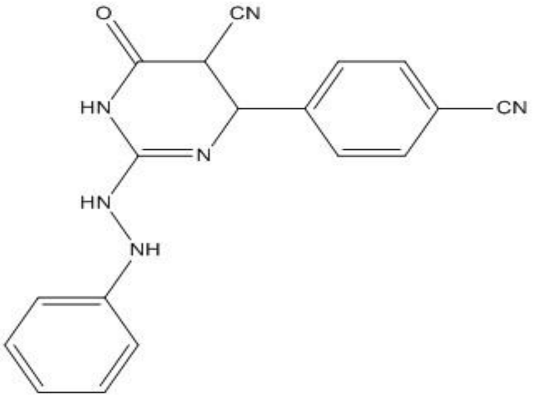

9

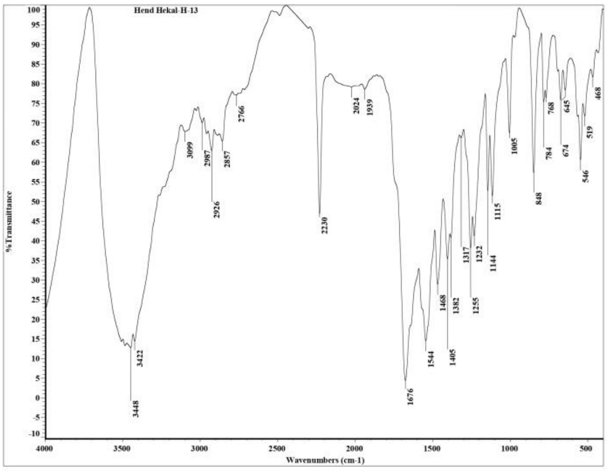

^{1}H-NMR Spectrum of (9):

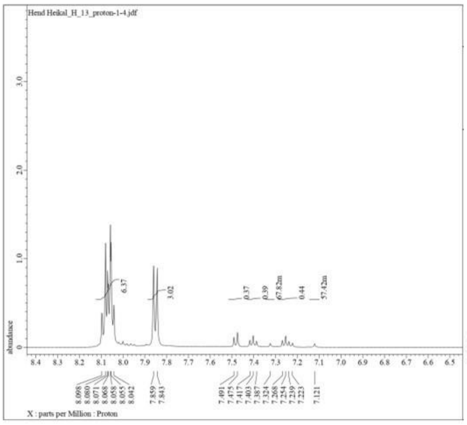
Hend Heikal_H_13_proton-1-4.jdf
abundance
X : parts per Million : Proton

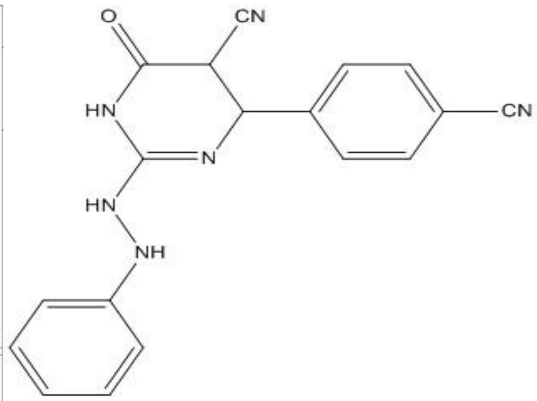
O
CN
HN
N
CN
HN
NH

9

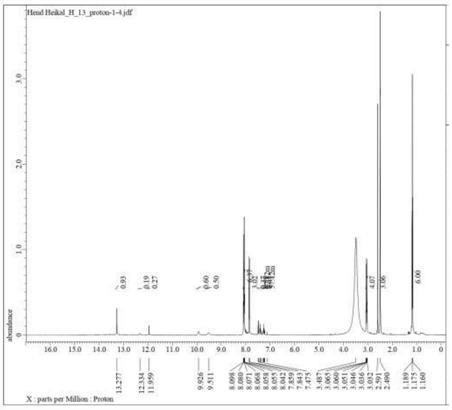
Hend Heikal_H_13_proton-1-4.jdf
abundance
X : parts per Million : Proton

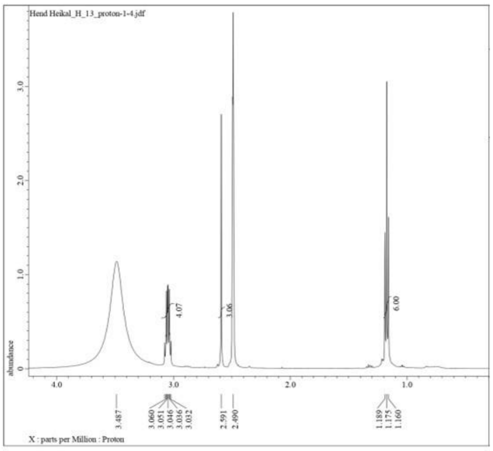
Hend Heikal_H_13_proton-1-4.jdf
abundance
X : parts per Million : Proton

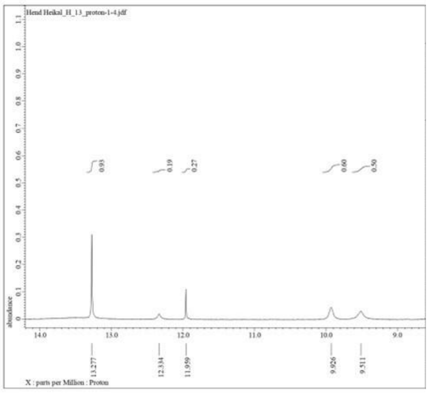
Hend Heikal_H_13_proton-1-4.jdf
abundance
X : parts per Million : Proton
