# Supplementary figures and images for: Innovation of eco-friendly TiO2 nano catalyst for new pyrimidine carbonitiriles candidates, assessed for significant antioxidant activity, anti-inflammatory effects, and by insilico studies
Source: PLoS One. 2025 May 29;20(5):e0313959. doi: 10.1371/journal.pone.0313959 (PMC12121771; doi:10.1371/journal.pone.0313959)

## Slide 1
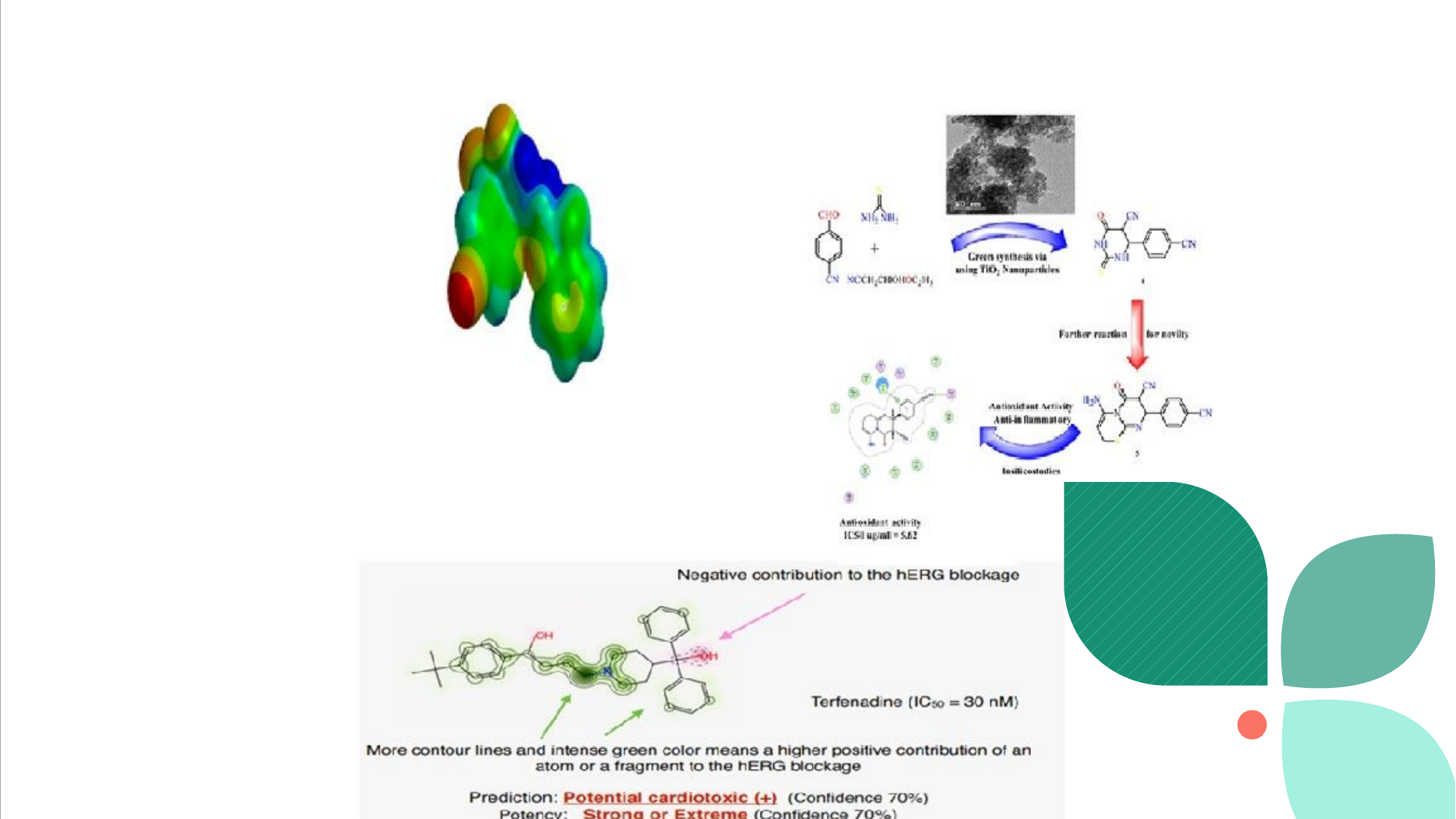

Supplement: S1 Graphical Abstract — (PPTX) [file pone.0313959.s010.pptx]
